# Beneficial impact of acquired AmpC β-lactamases on bacterial fitness and pathogenicity: a new paradigm

**DOI:** 10.1128/mbio.00088-26

**Published:** 2026-04-13

**Authors:** Otávio Hallal Ferreira Raro, Charlotte Michaux, Andrea Endimiani, Jorge Larios, Claudia Aldeia, Edgar I. Campos-Madueno, Jacqueline Findlay, Patrice Nordmann, Vincent Cattoir, Laurent Poirel

**Affiliations:** 1 Medical and Molecular Microbiology, Faculty of Science and Medicine, University of Fribourg 98839, Fribourg, Switzerland; 2 Department of Bacteriology, Rennes University Hospital 36684, Rennes, France; 3 UMR_S1230 Inserm BRM, University of Rennes 27079, Rennes, France; 4 National Reference Center for Antimicrobial Resistance (lab 'Enterococci'), Rennes University Hospital 36684, Rennes, France; 5 Institute for Infectious Diseases (IFIK), University of Bern 87618, Bern, Switzerland; 6 BioNanomaterials, Adolphe Merkle Institute, Adolphe Merkle Institute, University of Fribourg 311305, Fribourg, Switzerland; 7 Swiss National Reference Center for Emerging Antibiotic Resistance (NARA), University of Fribourg 27211, Fribourg, Switzerland; Instituto de Biologia Molecular y Celular de Rosario, Rosario, Santa Fe, Argentina

**Keywords:** AmpCs, fitness, pathogenicity

## Abstract

AmpC-type β-lactamases share structural similarities with penicillin-binding proteins, supporting the view that both enzyme families evolved from a common ancestor. Considering the widespread dissemination of acquired AmpCs in Gram-negative bacteria, we hypothesize that, besides their established role in β-lactam resistance, they may also influence essential bacterial mechanisms. Thus, this study aimed to elucidate whether AmpCs modulate bacterial physiology and fitness. *Escherichia coli* MG1655 was used as the model organism to assess the impact of clinically relevant acquired AmpCs (CMY, DHA, ACC, and FOX variants) on growth, competition, motility, biofilm formation, and pathogenicity, coupled with transcriptomic profiling. We demonstrated that certain CMY variants (notably CMY-42 and CMY-145) confer an unexpected *in vitro* growth advantage, whereas CMY-2 enhances pathogenicity *in vivo* by inducing flagellar production. These findings establish a new paradigm in which some AmpCs act as both resistance determinants and regulators of bacterial fitness and virulence, contributing to the ecological success of Enterobacterales.

**IMPORTANCE:**

Although β-lactamases have always been considered enzymes involved in resistance to β-lactam antibiotics, they also have to be considered as playing a role in reshaping bacterial physiology. By studying a series of class C β-lactamases, we uncovered an unexpected dual role. Besides conferring resistance to β-lactams, they also impact bacterial growth, motility, and pathogenicity through modulation of flagellar biosynthesis. Strikingly, while CMY-42- and CMY-145-producing recombinant *Escherichia coli* strains showed enhanced *in vitro* fitness growth by reducing energy-costly flagellar production, production of CMY-2 (the most commonly identified acquired AmpC β-lactamase identified in Enterobacterales) restored flagella and increased virulence *in vivo*. This work reframes β-lactamases as metabolic regulators influencing ecological success, offering new insight into how resistance determinants promote the spread and clinical impact of Enterobacterales.

## INTRODUCTION

β-Lactamases are enzymes able to compromise the ability of β-lactams to inhibit the growth of bacteria (1). They hydrolyze β-lactam antibiotics by cleaving the β-lactam ring, the essential component of those antibiotic molecules. Upon cleavage, the β-lactam is partially or totally inactivated, and its antibacterial activity is subsequently partially or entirely lost. An extensive list of β-lactamases has been identified so far, and they are classified either depending on their hydrolysis spectrum or their amino-acid sequence (2). The most commonly used nomenclature system is based on molecular structure; hence, some motifs present in the secondary structure of the amino-acid protein allow a classification dividing the β-lactamases into four main subgroups defined as Ambler classes A to D. Classes A, C, and D correspond to serine-active enzymes, whereas class B β-lactamases are metallo-enzymes requiring one or two active-site zinc ions for their hydrolytic activity (3).

Among the most commonly identified acquired β-lactamases conferring resistance to broad-spectrum cephalosporins in Gram-negative clinical isolates are the Ambler class A CTX-M enzymes (4–6) and the class C (also named AmpC) CMY-type enzymes (7). Noteworthy, AmpCs are naturally produced by most Gram-negative species, including clinically relevant Enterobacterales species (*Enterobacter cloacae, Klebsiella aerogenes, Morganella morganii, Serratia marcescens, Hafnia alvei,…*), as well as *Acinetobacter baumannii* and *Pseudomonas aeruginosa* species. In those latter isolates, the so-called *ampC* genes are chromosomally located, and the expression of these genes is often negatively regulated by a LysR-type regulator whose gene is located upstream of the *ampC* (8, 9). Noteworthy, acquisition of *ampC* is commonly observed among *Escherichia coli*, *Klebsiella pneumoniae,* and *Salmonella enterica* isolates. In particular, the *bla*_CMY_, *bla*_DHA_, *bla*_ACC_, and to a lesser extent *bla*_FOX_ genes are commonly found as plasmid-borne in those species, representing a source of acquired resistance to broad-spectrum cephalosporins, and ultimately to carbapenems when associated with major permeability defects (9). Interestingly, most of those genes commonly identified as acquired in Enterobacterales originate from the chromosome of other Enterobacterales. Hence, *bla*_CMY_ originates from *Citrobacter freundii, bla*_DHA_ from *M. morganii*, *bla*_ACC_ from *H. alvei*, although *bla*_FOX_ originates from *Aeromonas allosaccharophila* (10–13).

Owing to their wide distribution as intrinsic and chromosomally located genes in the majority of bacterial species, and the fact that these intrinsic genes are often weakly expressed in their natural hosts, it may be questioned if AmpC-encoding genes might play an additional role in the overall metabolism of the corresponding bacterial host, besides their ability to confer resistance to β-lactam antibiotics. In particular, the putative role of AmpC β-lactamases (AmpCs) in the peptidoglycan biosynthesis has been speculated, owing to the relative similarities in terms of protein structures and hydrolytic properties between AmpCs and penicillin-binding proteins (14), which are essential enzymes responsible for peptidoglycan assembly and the primary targets of β-lactam antibiotics. It is currently accepted that those two groups of enzymes derive from a common ancestor (15, 16).

Few studies have investigated the putative interplays between the production of AmpCs and overall bacterial fitness (ability to grow, virulence, and pathogenesis), as well as structural morphology. A recent study performed with *P. aeruginosa* as the model organism showed that the overproduction of its intrinsic chromosomally encoded AmpC (cAmpC), resulting from its corresponding gene overexpression (itself induced by impaired peptidoglycan recycling machinery), produced a negative effect on the virulence of those mutants (17). Another study highlighted that production of the cAmpC of *Enterobacter cloacae* in *Salmonella enterica* serotype Typhimurium had deleterious effects on its virulence features (18). Recently, Colquhoun et al. showed that the overproduction of the ADC-like cAmpC of *A. baumannii* had a significant impact on cell physiology, affecting the level of L,D-transpeptidase activity, and that overproduction of this AmpC had a deleterious effect on its virulence (19).

Bacterial fitness cost, that can be fundamentally defined as reduced growth rate, may result from modifications of enzymes through mutations, disruption of metabolic pathways through inactivation or loss of essential genes, or from the additional energy required for the expression or overexpression of constitutive or acquired genes, respectively. Therefore, fitness cost may be observed when bacteria acquire resistance to antibiotics through genetic mutations (20, 21).

Another well-known genetic event that may lead to a significant fitness cost corresponds to the acquisition (and subsequent maintenance and replication) of plasmids, the latter requiring energy for the bacterial cell that can impact some other metabolic capacities (22, 23). These negative consequences in terms of energetic cost on the growth rate have been shown to be related to the replication of the plasmid, but also at the lag phase, in relation to its acquisition (24). Thus, acquired resistance to antibiotics through plasmid gene acquisition is impactful for the bacterial fitness (20, 21).

In this study, we aimed to elucidate whether acquired AmpCs, beyond conferring resistance to β-lactam antibiotics, could also modulate bacterial physiology and fitness.

## RESULTS AND DISCUSSION

### Impact of CMY-type β-lactamases on the bacterial growth performances

Growth curves measurements were performed in parallel and under the same conditions (including agitation). CMY-2, CMY-42, and CMY-145 were the three representative AmpC enzymes initially selected in our study, because CMY-2 is the most predominant plasmid-encoded AmpC β-lactamase detected worldwide in Enterobacterales, and the two other variants have recently been increasingly reported, contributing to the decreased susceptibility to aztreonam-avibactam of *E. coli* isolates (7, 25–27). Thus, isogenic *E. coli* recombinant strains producing CMY-2, CMY-42, and CMY-145 (also corresponding to the variants currently predominating in our isolate collection at the Swiss National Reference Center for Emerging Antibiotic Resistance) were generated. Two control strains were used for comparison: a recombinant strain harboring an empty vector and the wild-type (WT) host strain lacking any plasmid.

**Fig 1:**
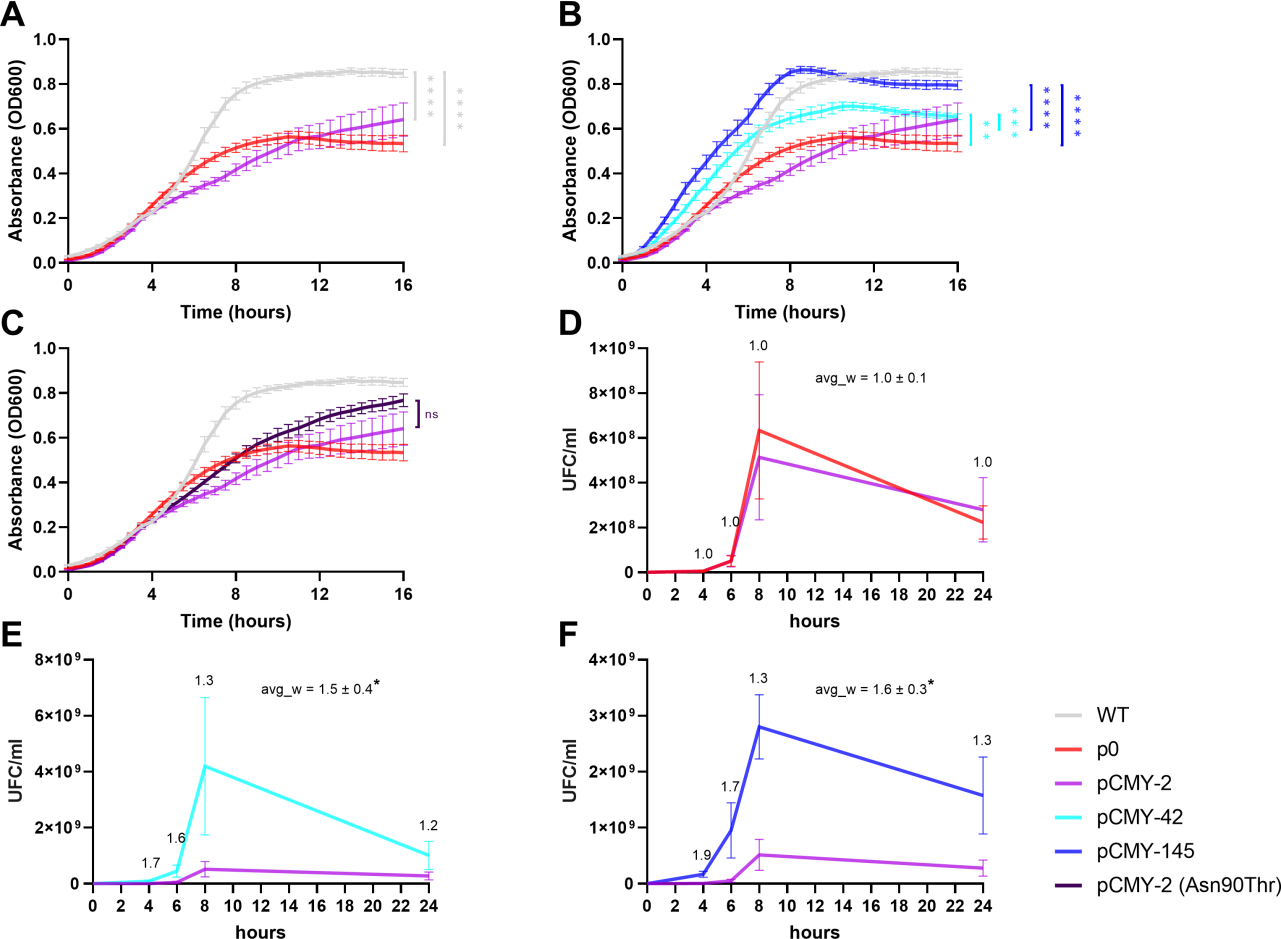
Measurement of growth capacities of the WT and recombinant isogenic *E. coli* isolates. (**A**) Growth curves evidencing the negative impact on the growth rate caused by the insertion of the plasmid. (**B**) Demonstration of the effect of compensation observed when pCMY-42 and pCMY-145 are present. (**C**) Demonstration of the lack of significant activity observed when the Asn90Thr substitution was inserted in CMY-2. (**D**) Competition assay showing no relative fitness discrepancies between p0 and pCMY-2. (**E**) Competition assay showing relative fitness advantage for pCMY-42 over pCMY-2. (**F**) Competition assay showing relative fitness advantage for pCMY-145 over pCMY-2. Data are shown as mean ± standard error of mean (SEM). ns, non-significant; *, *P*-value ≤ 0.05; **, *P*-value ≤ 0.01; ***, *P*-value ≤ 0.001; ****, *P*-value ≤ 0.0001. For panels **D**, **E**, and **F**, numbers show relative fitness in each time point (4 h, 6 h, 8 h, and 24 h); Avg_w shows global average relative fitness ± SEM.

First, we observed that replication of the pTOPO plasmid lacking any β-lactamase gene had a negative impact on the growth rate compared to the WT strain (WT: 1.4 × > p0), which confirms previous findings (Fig. 1A) (20, 21). Once transformed with a recombinant pTOPO plasmid encoding the CMY-2 β-lactamase, no difference was observed with the isogenic strain harboring a similar-in-size plasmid not encoding CMY-2, still evidencing a negative impact of the plasmid carriage on the growth rate (WT: 1.5 × > pCMY-2) (Fig. 1A). By contrast, the growth rate of the CMY-42 (point mutant Val211Ser derivative of CMY-2 [Fig. S1]) was significantly higher than the CMY-2 isogenic strain (pCMY-42: 1.3 × > pCMY-2), the former rate being in the same range as the one observed with the recipient strain lacking any plasmid. Furthermore, an even higher growth rate (pCMY-145: 1.6 × > pCMY-2) was observed with the isogenic strain producing CMY-145 (point mutant derivative of CMY-42, two amino-acid substitutions compared to CMY-2, namely Asn90Thr and Val211Ser [Fig. 1B; Fig. S2]). Those results indicated that production of the CMY-42 and CMY-145 specific variants provided a fitness advantage to *E. coli* under *in vitro* conditions compared to the same recipient strain harboring an “empty” plasmid, but most of all that production of CMY-2 had a deleterious effect on the growth *in vitro*.

To evaluate whether the Asn90Thr substitution observed in CMY-145 compared to CMY-42 was possibly playing a role in the observed differences, site-directed mutagenesis experiments were conducted to introduce an Asn90Thr substitution in CMY-2, but no significant effect was observed in terms of growth curves when compared to the two isogenic *E. coli* recombinant strains producing CMY-2 and its derivative (Fig. 1C). This suggested that the differences observed with CMY-42 and CMY-145 compared to CMY-2 were mainly related to the Ser residue at position 211 and that the Thr residue at position 90 was potentializing this feature only once Ser211 was present.

### Mutation of Ser64Arg in the catalytic site of AmpC impairs growth capacity of pCMY-145

Considering that the implication of CMY-like variants in the bacterial growth of the corresponding producers remained unexplained, we aimed to evaluate whether this might be related to a specific enzymatic property, or rather to a structural conformation of the CMY protein that might interact with one or several specific targets. Since the catalytic activity of serine β-lactamases in general, and CMY-type β-lactamases in particular, is known to be fully dependent on the presence of a Ser residue at position 64, our strategy was to substitute this specific amino acid to impair the enzymatic activity of the corresponding mutant. Recombinant plasmid pCMY-145-Ser64Arg was therefore generated using a site-directed mutagenesis strategy targeting the CMY-145 variant, and the corresponding *E. coli* transformant was tested for its growth capacities.

First, susceptibility testing confirmed the complete loss of catalytic activity of the CMY-145 derivative mutant since a full susceptibility to all β-lactams was observed for the corresponding recombinant strain (Table S1). Then, growth curve measurements showed a significant disadvantage (*P*-value ≤ 0.0001) of the strain producing the CMY-145-Ser64Arg variant compared to CMY-145 (pCMY-145: 1.8 × > pCMY-145-Ser64Arg) (Fig. S1). This allowed us to speculate that the fitness advantage observed through the production of the CMY-145 variant *in vitro* was likely resulting from a productive catalytic activity. Moreover, production of the CMY-145-Ser64Arg mutant protein added to the energy cost on top of the plasmid replication for that recombinant strain.

### Further assessment of fitness advantages conferred *in vitro* by CMY-42 and CMY-145 β-lactamases

To further confirm those observations obtained through growth curve measurements, competition assays including only two strains in each assay were performed to assess whether some growth advantages could be observed. They showed that the CMY-42- and CMY-145-producing *E. coli* recombinant strains were respectively outcompeting the CMY-2 producer (pCMY-42 relative fitness: 1.5 × > pCMY-2 and pCMY-145 relative fitness: 1.6 × > pCMY-2) (Fig. 1E and F; Fig. S3). They also outcompeted the *E. coli* (p0) recombinant strain to the same extent, no significant difference being observed between those strains carrying pCMY-2 and p0, respectively (Fig. 1D).

These results further suggested that the Ser residue at position 211 was providing a particular feature to CMY-type β-lactamases, positively impacting the fitness of the host strain under *in vitro* conditions, but most of all that substitution with a Thr at position 211 was deleterious. Of note, a total of 223 CMY variants have been so far identified according to databases (http://bldb.eu/alignment.php?align=C:CMY), and 16 of them possess a Ser residue at position 211 (Fig. S2).

### Other AmpCs may also impact the bacterial growth performances of *E. coli*

Considering the notable observations associated with certain CMY variants, being the most commonly acquired AmpCs among enterobacterial isolates (particularly *E. coli* and *K. pneumoniae*), we aimed to investigate whether other clinically relevant AmpCs might also influence bacterial fitness. Therefore, a series of AmpC-encoding genes frequently identified as plasmid-borne and acquired in *E. coli* were cloned and expressed in *E. coli* K12*.* These genes encoded DHA-1, ACC-1, and FOX-5. These three β-lactamases, although being distantly related in terms of protein secondary structure (40%–50% amino-acid identity between each other), share very similar β-lactam hydrolysis profiles compared to CMY (overall same substrates hydrolyzed or spared, and similar sensitivities or resistance to β-lactamase inhibitors). By testing those different recombinant *E. coli* strains in comparison with *E. coli* (p0), neither growth curve measurements nor competition assays revealed any significant difference (Fig. S4 and S5).

Considering that those different AmpCs did not possess the Ser residue identified at position 211 in CMY-42 and CMY-145, but all harbored a Val residue as in the CMY-2 sequence when aligning all the AmpC variants, site-directed mutagenesis experiments were performed to replace the Val residue with a Ser at position 211 (28). Our goal here was to determine whether the benefit observed *in vitro* with CMY-42 or CMY-145 compared to CMY-2 could also be observed for other AmpC-type enzymes.

Interestingly, by performing growth curve measurements or competition assays, a similar effect was indeed observed for the DHA-1-Ser211 and the FOX-5-Ser211 mutant enzymes, both outcompeting their respective original counterparts exhibiting a Val at position 211. The differences observed in terms of growth rates and through competition assays were 1.5× and 1.4× for growth rate and 1.3× and 1.8× for relative fitness for pDHA-1-Ser211 and pFOX-5-Ser211 in comparison with the DHA-1 and FOX-5, respectively (Fig. S4 and S5). By contrast, no significant difference was observed between the ACC-1-producing *E. coli* recombinant strain and its mutated counterpart.

### Global transcriptomic analysis revealed that flagellar biosynthesis is affected by the production of CMY-2 in *E. coli*

**Fig 2:**
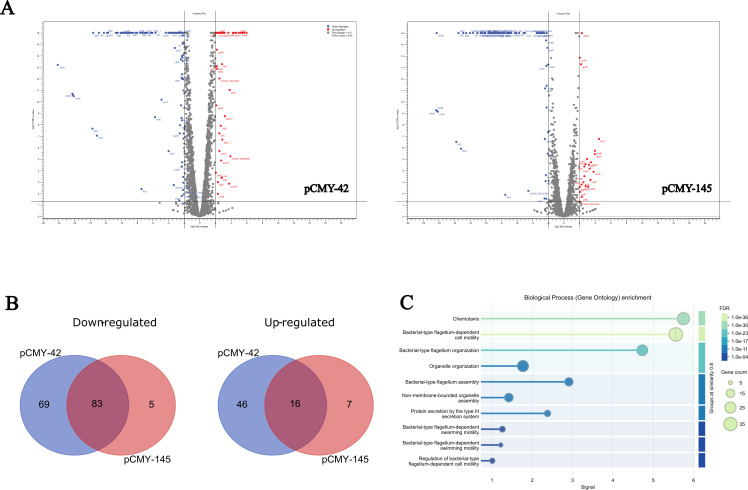
RNA transcriptomic analysis. (**A**) Volcano plots representing graphical data visualization of 4,351 genes present in recombinant isogenic *E. coli* isolates, showing the expression differences (≥2 log2 fold) and statistical significance (*P*-value ≤ 0.05) when comparing pCMY-42 or pCMY-145 with pCMY-2. (**B**) Venn diagrams showing isogenic recombinant pCMY-42 and pCMY-145 down- or upregulated genes when compared with pCMY-2. (**C**) Gene ontology enrichment of genes downregulated in CMY-42 and CMY-145 compared to CMY-2. Biological process terms enriched among downregulated genes were used. Circle size indicates the number of downregulated genes; signal is defined as a weighted harmonic mean between the observed/expected ratio and −log; FDR < 0.05.

Considering the unexplained phenotypic growth discrepancies observed between the CMY-2-producing *E. coli* on one hand, and the CMY-42- and CMY-145-producing *E. coli* on the other hand, we initiated a comparative RNA transcriptomic analysis for these different but isogenic recombinant strains. Transcriptomic assays were performed on four different isogenic *E. coli* recombinant strains, namely the same *E. coli* strain carrying (i) the p0 empty vector, (ii) the recombinant plasmid carrying *bla*_CMY-2_, (iii) *bla*_CMY-42_, and (iv) *bla*_CMY-145_. Although hundreds of genes were found to be up- or downregulated in all CMY-producing strains in comparison with the same strain carrying the empty vector (no β-lactamase gene) (Table S2), a significant (>2 log_2_; false discovery rate [FDR] < 0.05) fold change (up or down) was observed for 214 genes when comparing the strain producing CMY-42 vs the strain producing CMY-2, and 111 genes when comparing strain producing CMY-145 vs strain producing CMY-2 (Table S2; Fig. 2A).

Then, we performed a restrictive analysis by considering only those genes whose expression was significantly up- or downregulated in both the CMY-42- and CMY-145-producing strains, but not in the CMY-2-producing strain. We could identify a total of 83 downregulated genes and a total of 16 upregulated genes in both CMY-42 and CMY-145 when compared with CMY-2 (Fig. 2B). Although the log_2_ fold change of the upregulation was found to vary from 2.3 to 6.0, higher log_2_ fold changes were found for the downregulated genes, varying from −2.1 to −18.1 (Table S3).

Among the genes whose expressions were downregulated compared to the CMY-2 producer, we selected those with the higher fold difference (> log_2_ −9) to filter a potential background not related to the observed phenotype, ending up with a batch of 25 genes. Noteworthy, the majority (23 out of 25) were common for the CMY-42 and the CMY-145 producing strains, and those 23 genes were subsequently retained for our analysis. Interestingly, the vast majority (17 out of 23) of those genes encoded proteins related to flagellar biosynthesis. Briefly, flagellar genes of *E. coli* are organized in a hierarchical regulatory network of three classes based on their order of expression and common transcriptional regulation (29, 30). This network encodes more than 40 proteins that are necessary for flagellar motility and chemotaxis (31). Particularly, class II genes are those encoding the components related to motility and were found to be the most affected in our transcriptomic assays (31).

This strongly suggested that those specific CMY variants, in addition to a broad-spectrum β-lactam resistance, provided a fitness advantage to *E. coli* under *in vitro* conditions (under agitation) that might be related to decreased flagellar production, consequently decreased energy cost, increased DNA replication, eventually leading to a higher fitness when considering growth rate as a main feature.

The selected genes included the *flhD* and *flhC* (being considered as the *flhCD* tandem) (both genes being downregulated at a −13.1 log_2_ fold change) encoding the so-called master regulator FlhD_4_C_2_ corresponding to a heterohexamer controlling the expression of the entire flagellar regulons (32, 33). The flagellar biosynthesis is known as a three-tiered cascade, compounded by the master regulators (representing class I genes), known to negatively regulate the expression of the so-called class II genes such as *fliFGHIJK* and *flgBCDEFGHIJ* encoding structural and assembly proteins required for the biosynthesis of the hook-basal body of the flagellum. In addition, the genes *fliA* and *flgM*, respectively encoding FliA (sigma factor σ28) and FlgM (anti-sigma factor) regulators, are responsible for induction or repression of class III genes such as *fliC*, *flgKL*, *motAB*, and *cheAW*, those latter encoding flagellar rotation and chemotaxis, as well as structural components of the flagellum (34, 35). Considering that FlhD_4_C_2_ is the master regulatory protein of these class II genes, and therefore indirectly of the class III genes, completing the flagellum biosynthesis cascade, our data are consistent with the literature and would suggest that FlhD_4_C_2_ is likely the key feature in the observed phenotype (fitness advantage).

Additionally, we performed a gene ontology (GO) enrichment analysis of genes downregulated in CMY-42 and CMY-145 compared with CMY-2. The results confirmed the presence of a strong over-representation of processes associated with motility and flagellar function. The most significantly enriched terms were bacterial-type flagellum-dependent cell motility (GO: 0071973; FDR = 2.51 × 10⁻³⁶; 36 genes), chemotaxis (GO: 0006935; FDR = 2.75 × 10⁻³¹; 29 genes), and bacterial-type flagellum organization (GO: 0044781; FDR = 7.08 × 10⁻²⁴; 23 genes). Additional enriched processes included bacterial-type flagellum assembly (GO: 0044780; FDR = 3.86 × 10⁻¹³; 14 genes), organelle organization (GO: 0006996; FDR = 3.06 × 10⁻¹³; 24 genes), and non-membrane-bounded organelle assembly (GO: 0140694; FDR = 3.05 × 10⁻⁰⁸; 15 genes) (Fig. 2C). These results reinforce a coordinated downregulation response observed in recombinant strains producing CMY-42 and CMY-145 for the genes required for flagellum assembly, motility, and chemotaxis, suggesting a potential reduction in motility-related functions, especially related to the impairment of the flagellar biosynthesis cascade when compared to CMY-2.

### RT-qPCR assays showed a similar impact of production of DHA-1 and FOX-5 on flagellar biosynthesis in *E. coli*

**Fig 3:**
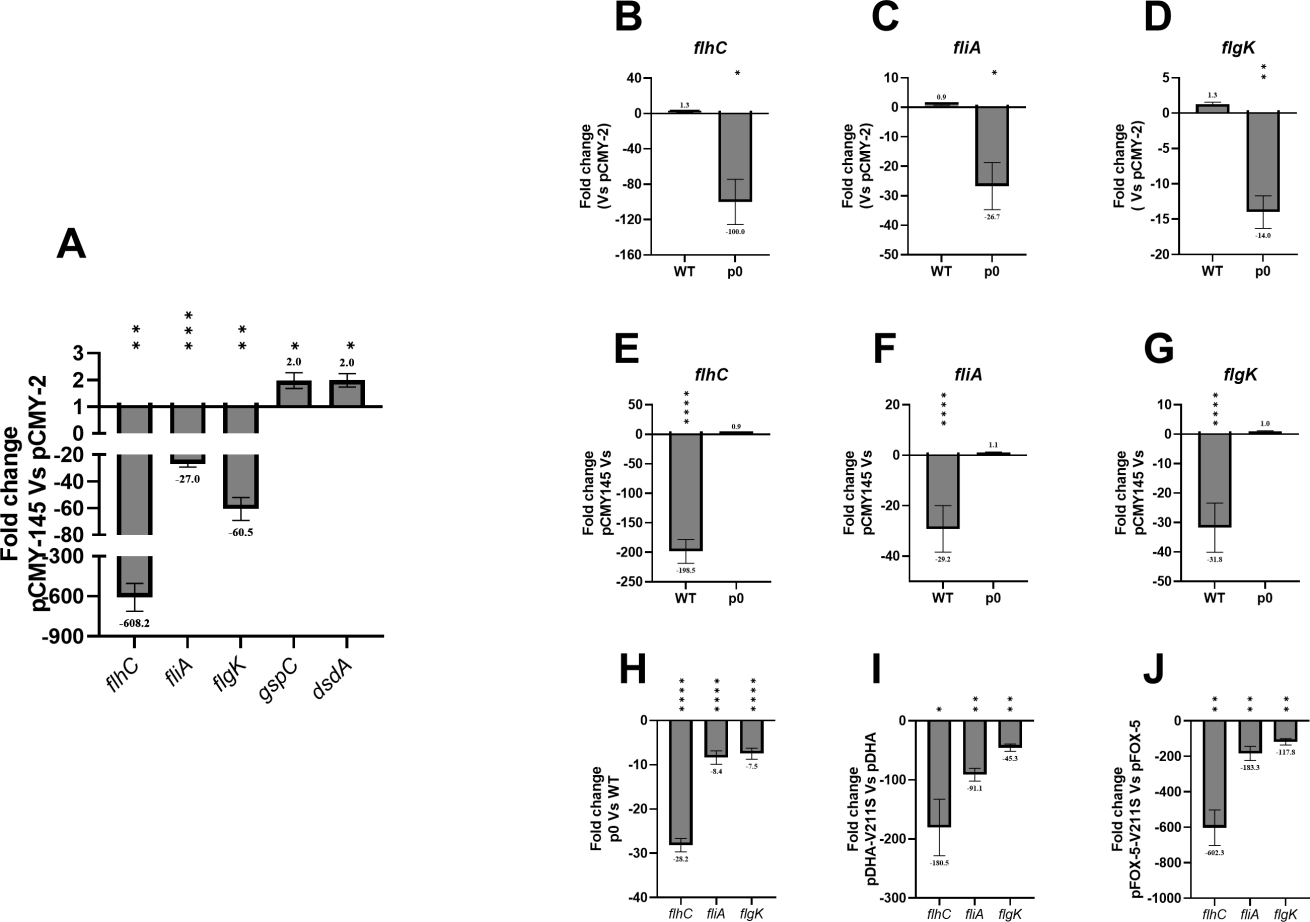
RT-qPCR. (**A**) Comparison of the relative expression of pCMY-2 against pCMY-145 for the genes *flhC*, *fliA*, *flgK*, *gspC,* and *dsdA*, confirming transcriptomic results. (**B**) *flhC* expression for pCMY-2 against WT and p0. (**C**) *fliA* expression for pCMY-2 against WT and p0. (**D**) *flgK* expression for pCMY-2 against WT and p0. (**E**) *flhC* expression for pCMY-145 against WT and p0. (**F**) *fliA* expression for pCMY-145 against WT and p0. (**G**) *flgK* expression for pCMY-145 against WT and p0. (**H**) *flhC*, *fliA*, *flgK* expression for p0 against WT. (**I**) *flhC*, *fliA*, *flgK* expression for pDHA-V211S against pDHA. (**J**) *flhC*, *fliA*, *flgK* expression for FOX-5-V211S against FOX-5. Results observed for pCMY-145 can be extrapolated to pCMY-42. Data are shown as mean ± SEM. *, *P*-value ≤ 0.05; ** *P*-value ≤ 0.01; ***, *P*-value ≤ 0.001; ****, *P*-value ≤ 0.0001**.**

In full agreement with the transcriptomic results, a downregulation of the *flhC*, *fliA*, and *flgK* genes was observed when comparing the CMY-145 producer to the CMY-2 one. On the other hand, the expression of *gspC* and *dsdA* genes was upregulated upon CMY-145 production when comparing with CMY-2 (Fig. 3A). The same level of expression was found for *flhC*, *fliA*, and *flgK* genes when comparing the CMY-2 producer and the WT (Fig. 3B through D). The p0 strain showed a downregulation of *flhC*, *fliA*, and *flgK* genes when comparing with the recombinant strain carrying the CMY-2 (Fig. 3B through D). These findings reinforce that the simple presence of the plasmid on its own (p0) produced a downregulation in genes responsible for the flagellar biosynthesis, and that the addition of CMY-2 compensated for that loss by upregulating those genes.

Of note, the expressions of the *flhC*, *fliA*, and *flgK* genes were found to be downregulated upon production of CMY-145 when comparing to the WT strain, but at similar levels when compared to the recombinant strain carrying p0 (Fig. 3E through G). In addition, the expression of genes involved in the flagellar biosynthesis was downregulated when comparing the *E. coli* (p0) recombinant to its WT isogenic counterpart, confirming once again that the introduction of the plasmid by itself impaired this cascade (Fig. 3H).

Interestingly, a significant downregulation of the genes involved in the flagellar biosynthesis (*flhC*, *fliA*, and *flgK*) was also observed upon production of the mutated AmpC variants DHA-1-V211S and FOX-5-V211S when compared with their respective counterparts upon production of DHA-1 and FOX-5 (Fig. 3I and J), respectively. Hence, the implication of other AmpCs besides CMY-2 in the regulation of the flagellar biosynthesis cascade was also proven here.

### Compensated flagellar biosynthesis after plasmid insertion is likely not mediated by direct binding of CMY-2 to the FlhD₄C₂ master regulator sequence

Results obtained from the electrophoretic mobility shift assay (EMSA) experiments showed that there was no shift in the migration pattern of the *flhD* amplicons, including or not the promoter sequences, indicating a lack of binding between the protein tested and the DNA, including the upstream located promoter sequences. This result was repeatedly observed independently either of the variant, CMY-2, CMY-42, or CMY-145 (L. Poirel, unpublished data).

### Motility assays further suggest the involvement of flagellar biosynthesis dysregulation upon production of certain CMY β-lactamases in *E. coli*

**Fig 4:**
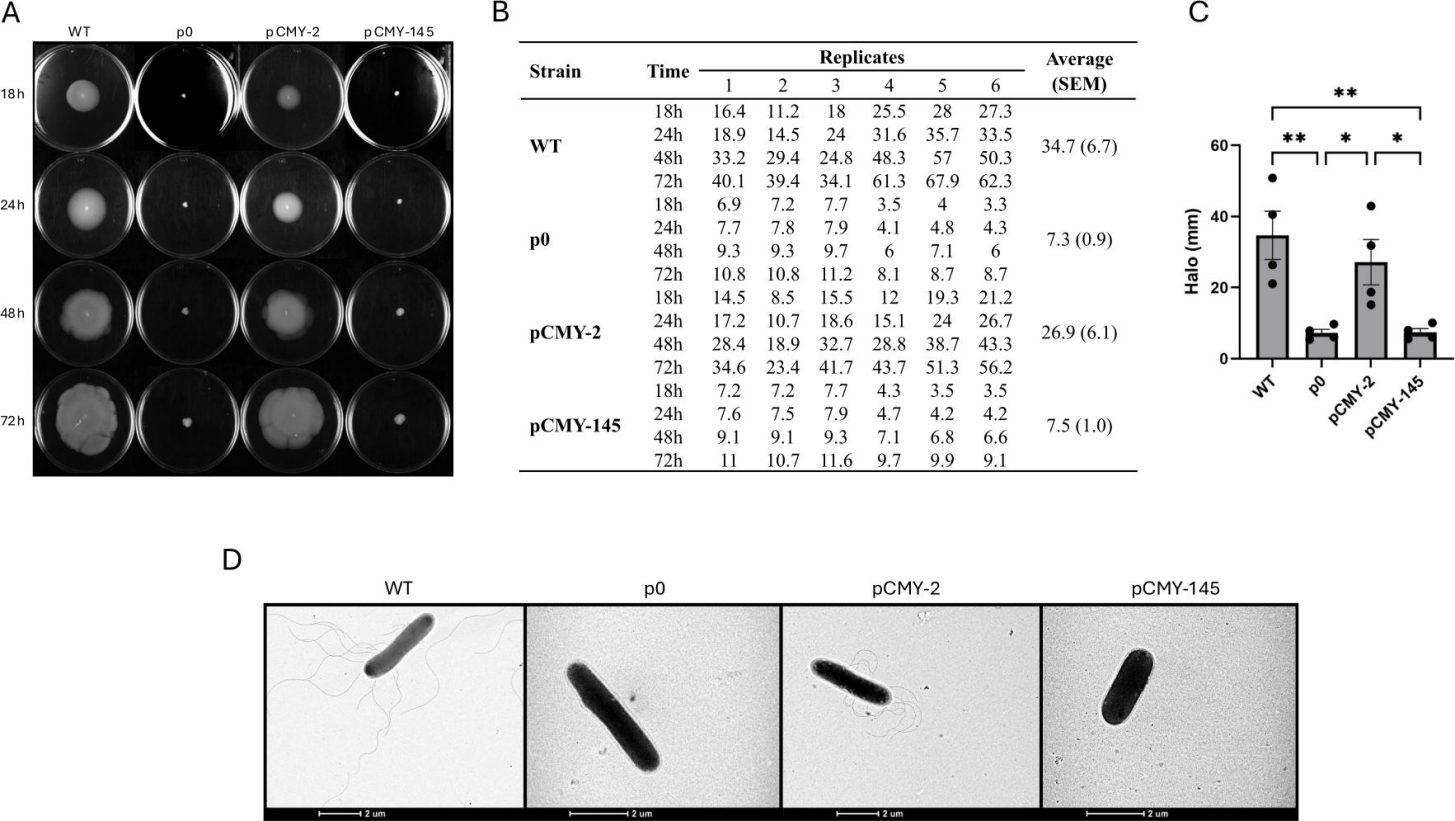
Motility assay in soft LB agar and transmission electron microscopy. (**A**) Colony swimming demonstrates halo growth in 18 h, 24 h, 48 h, and 72 h. (**B**) Table with the halo diameter measured for all six replicates. (**C**) Bar graph demonstrating halo differences. Data are shown as mean ± SEM. *, *P*-value ≤ 0.05; **, *P*-value ≤ 0.01. Results for pCMY-42 are in the same range as for pCMY-145. (**D**) Transmission electron microscopy evidencing the presence or absence of flagella in the cell structure of the WT and isogenic *E. coli* strains. Pictures were taken using a Tecnai G2 Spirit bioTwin transmission electron microscope at 120 kV equipped with a WA Veleta camera. Scale bar corresponds to 2 µm. Images observed for pCMY-145 can be extrapolated to pCMY-42.

Results showed that the CMY-2-producing *E. coli* could massively expand over the agar plate (halo: 26.9 ± 6.1 mm), evidencing an invasive behavior, in total contrast with the CMY-42- or CMY-145-producing isogenic recombinant *E. coli* strains, the latter forming only a small colony (7.5 ± 1 mm) (Fig. 4A through C). Those observations were fully in line with a significant discrepancy in terms of flagellar production.

### Production of CMY variants impacts the biofilm formation

Although the replication of p0 was shown to negatively impact the biofilm formation compared to the WT strain without plasmid, the CMY-2 production was shown to compensate for this negative impact, while the production of CMY-145 led to a twofold higher production of biofilm compared to that of CMY-2 (Fig. S6).

### Flagellar production is lost upon plasmid acquisition, recovered upon production of CMY-2, but not of CMY-145

Although flagellar production was indeed well observed in the WT *E. coli* strain, it was almost abolished once harboring the empty vector, therefore assessing the dysregulation generated by the plasmid acquisition, regardless of any β-lactamase production (Fig. 4D). More interestingly, a significant discrepancy was observed between the CMY-2- and CMY-145-producing isogenic *E. coli* strains, showing a lack of flagellar production for the latter but a high level of flagellar production (comparable to the wild-type strain) for the former. Hence, CMY-2 production was a source of compensation for the plasmid introduction with respect to flagellar biosynthesis, and CMY-145 production, on the opposite, did not modify this phenotype, despite providing a fitness advantage *in vitro* under agitation. We could conclude that the dysregulation of the flagellar biosynthesis was a major metabolic feature explaining the phenotype discrepancies observed *in vitro*.

### Lack of growth discrepancies observed upon CMY variant production in the non-flagellated *K. pneumoniae* species

A *K. pneumoniae* WT strain and its isogenic recombinant counterparts were subjected to evaluation of their growth performance following a similar approach as for *E. coli.* Hence, recombinant *K. pneumoniae* strains producing either no β-lactamase (p0) or CMY-2, CMY-145, and CMY-42 variants were generated. As expected for such species that do not naturally produce flagella, neither replication of p0, pCMY-42, pCMY-145, nor pCMY-42 showed any effect on the growth of these isogenic strains, all of them exhibiting similar growth rates compared to the WT strain (Fig. S7).

### Deletion of the master regulator *flhDC* equalizes growth capacities of pCMY-2 to pCMY-145

To perform further validation of the *E. coli* MG1655 model used throughout this study, and to evaluate the influence of the *flhDC* gene cluster on growth performance, two strains from the KEIO collection were used, being either the WT (KEIO) or its mutant derivative exhibiting an inactive *flhD* gene (KEIO-ΔflhD) (36). Both isogenic strains were transformed either with p0, pCMY-2, pCMY-42, or CMY-145 to respectively generate the *E. coli* KEIO recombinant strains.

When testing the *E. coli* KEIO WT strain, replication of plasmids p0 or pCMY-2 generated decreased growth capacities, as evidenced in the former evaluations with *E. coli* MG1655 (Fig. S8A). In line with those previously obtained data, production of CMY-42 or CMY-145 was also beneficial for the strain showing a compensatory effect on the growth rate when compared with the KEIO-p0 and the KEIO-pCMY-2 recombinant strains (Fig. S8B).

When testing the *E. coli* KEIO-Δ*flhD* strain, first a higher growth rate was observed when compared to the *E. coli* KEIO-Δ*flhD*-p0, KEIO-Δ*flhD*-pCMY-2, KEIO-Δ*flhD*-pCMY-42, and KEIO-Δ*flhD*CMY-145 (Fig. S8C). Noteworthy, no difference in growth rates was observed between all *E. coli* KEIO-*flhD* recombinant strains producing CMY-2, CMY-42, or CMY-145 (Fig. S8C). Hence, these data further evidenced the critical role of the FlhD_4_C_2_ master regulator and the corresponding flagellar biosynthesis in the growth fitness of *E. coli* strains.

### Resistance to serum killing activity mediated by production of CMY-2, but not CMY-145

**Fig 5:**
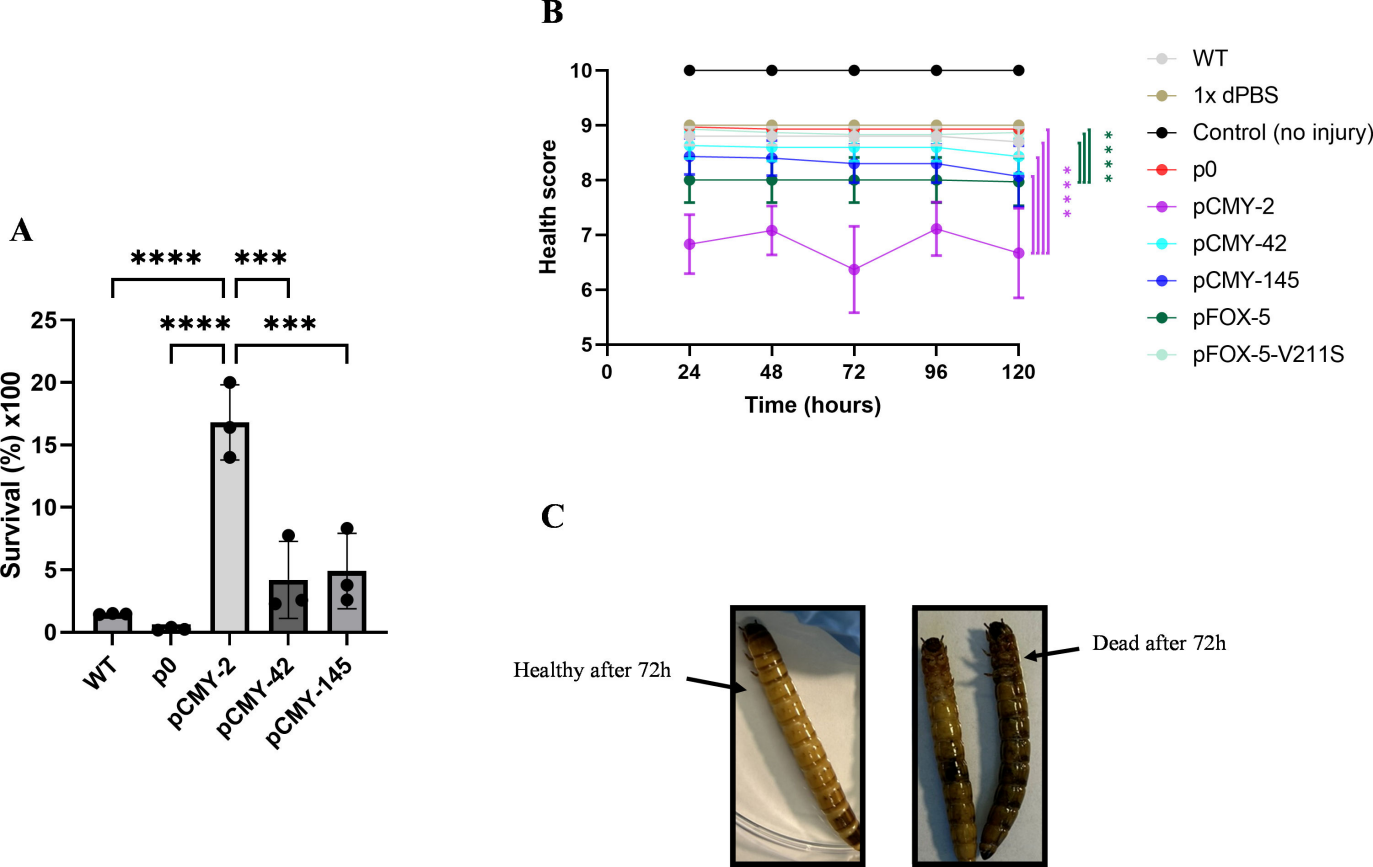
Serum-killing assays and *in vivo* larvae pathogenesis. (**A**) Survival percentage of the strains in 33% human serum. Data are shown as mean ± SEM. ***, *P*-value ≤ 0.001; ****, *P*-value ≤ 0.0001. (**B**) Health score of infected *Zophobas morio* larvae (*Zm*L) at different time points. The total health score of each treatment per time point is represented by the mean ± SEM. The significance level of the *P*-values for comparing pCMY-2 (purple vertical lines) against WT, p0, pCMY-42, and pCMY-145, and pFOX-5 (green vertical lines) against WT, p0, and pFOX-5-V211S are: ****, *P*-value ≤ 0.0001. Only significant results (*P* < 0.05) of the interactions between pCMY-2 and/or pFOX-5 against all other treatments, as determined by ordinal logistic regression, are shown in the plot. (**C**) Picture taken for demonstration of the differences in larvae states after 72 h of the experiment. Left-hand side: healthy *Zm*L. Right-hand side: dead *Zm*L infected with pCMY-2.

In fact, despite production of CMY-145 showing a fitness advantage under agitation, the CMY-2-producing recombinant strain showed higher resistance through *in vitro* serum killing assays performed without agitation (Fig. 5A). Interestingly, this higher resistance observed with pCMY-2 with respect to pCMY-145 was also observed with respect to p0 and to the WT *E. coli*. Thus, production of CMY-2 is likely advantageous in terms of pathogenicity for *E. coli* isogenic strains, taking into account that these serum killing assays were performed without shaking.

### Production of CMY-2 or FOX-5 enhances the pathogenicity of *E. coli* using an *in vivo* model

Considering the results of our different *in vitro* assays, highlighting that production of CMY-42 or CMY-145 provided a fitness advantage compared to CMY-2, followed by results of the *in vitro* serum killing assays showing a paradoxical advantage conferred by the production of CMY-2 compared to CMY-42 and CMY-145, an *in vivo* experimental model was considered.

Using the *Zm*L model and its scoring system (Table S4), we observed that the CMY-2-producing *E. coli* strain was much more pathogenic than its isogenic counterparts producing CMY-42 or CMY-145, respectively (Fig. 5B). Nonetheless, production of these two CMY variants also showed a deleterious impact on the larvae’s health compared to the same strain lacking β-lactamase production, but to a much smaller impact compared to the CMY-2 producer (Fig. 5B). We therefore clearly evidenced that production of CMY-2 was a significant factor increasing the pathogenicity of *E. coli* in this *in vivo* model. Figure 5C illustrates representative larval states, contrasting a healthy control *Zm*L with those that died by pCMY-2 infection after 72 h.

To evaluate whether those observations obtained *in vivo* for CMY-2 could be observed for another representative of the AmpC β-lactamase group, the pathogenicity of the FOX-5 and FOX-5-V211S producers was also compared using this larval model. It showed that production of FOX-5 also provided a significant pathogenesis advantage. Notably, the Val to Ser amino-acid substitution at position 211 in the FOX-5 sequence, which was shown to be a source of fitness advantage under *in vitro* and agitation conditions, was actually shown to be conversely disadvantageous under *in vivo* conditions.

### Conclusion

The role of β-lactamases, besides their ability to confer resistance to β-lactams, has been considered as possibly related to other features, but without clear indications of any putative additional function. Through the present study, we have evidenced a new paradigm, showing that some specific AmpCs contribute to a gain of fitness, once produced in *E. coli.* In our study, that gain was shown to compensate the fitness cost induced by the acquisition of a high-copy plasmid. Very importantly, our study showed that the fitness advantage evidenced upon production of given β-lactamase variants under *in vitro* conditions, hence using cultures performed under agitation, was conversely not evidenced when considering *in vivo* pathogenesis using an *in vivo* model or when static growth was applied, as through *in vitro* serum killing assays. By contrast, production of other β-lactamase variants for which no fitness advantage was evidenced under *in vitro* conditions was proven to be a significant pathogenesis booster when considering the *in vivo* model. Hence, the pathogenesis advantage was observed for the AmpCs CMY-2 and FOX-5. Notably, we acknowledge that expression levels from a high-copy number plasmid may be a limitation as it does not perfectly recapitulate chromosomal or low-copy expression contexts.

We could explain these paradoxical observations by identifying the main phenotypic feature responsible for this discrepancy, namely the flagellar production. Indeed, we showed that one significant metabolic response of the acquisition of a high-copy plasmid in the *E. coli* WT strain was to downregulate genes involved in flagellar biosynthesis, then almost abolishing phenotypically the flagellar production, as clearly evidenced through electron microscopy and motility assays. We might speculate that this abolishment induced a significant energy saving for the bacterial cell, consequently allowing a compensation in terms of growth rate under the used *in vitro* conditions, considering that mitigation of flagellar production is likely not significantly impacting the bacterial spread in those conditions. Indeed, considering that the bacterial flagellum is composed of more than 60 gene products, saving the corresponding biosynthetic energy expenditure, which was estimated to be ca. 2% of the total energy cost of a bacterial cell (37, 38), was very likely crucial. This is in line with observations reported by Plague et al. (39) who showed that truncation of flagellar genes leading to flagellar biosynthesis impairment and subsequent motility defect conferred a fitness advantage in their evolution experiment in *E. coli*. Interestingly, Ni et al. showed that the fitness cost of motility was primarily due to the biosynthesis of flagella (40), but the flagellar rotation itself was also considered costly (41).

Very surprisingly, we showed that production of CMY-2, DHA-1, or FOX-5 could restore the production of the flagella, which had a negative effect on the *in vitro* observed bacterial fitness but had a positive effect when considering the *in vivo* pathogenesis model. Although these results might be considered paradoxical, they are meaningful when considering the benefit of energy saving upon downregulation of the flagellar biosynthesis, which is counterbalanced by the fact that *in vitro* assays conducted under agitation minimize the role of the flagella. By contrast, considering that flagellar production enhances the bacterial pathogenesis by favoring the bacterial dissemination and the escape from the immune system appears to be meaningful, and this might be one reason why CMY-2 is epidemiologically successful (42, 43).

The way β-lactamase production interferes with the flagellar production remains partially clarified. We showed that the expression of the genes encoding all the steps of flagellar biosynthesis was upregulated upon production of CMY-2, DHA-1, and FOX-5, and that this upregulation was related to the overproduction of the FlhDC master regulator through an upregulation of the expression of the *flhC-flhD* tandem. Nevertheless, there was no significant difference observed in terms of expression level for the gene encoding HdeD, which is known to be a membrane regulator repressing the synthesis of flagella (37, 38), when comparing production of CMY-2 or CMY-42, respectively. The way how the different variants of AmpCs could interfere with the expression of such genes remains so far unexplained.

Noticeably, we showed that the mechanism sustaining the flagellar biosynthesis induction of CMY-2 was very likely resulting from its enzymatic activity rather than another structural feature. And we reinforced this hypothesis by showing that no binding was observed between *flhD* and CMY-2 through EMSA. Future work will be necessary to precisely identify how the interaction between the CMY variants and the master regulator controlling the flagella production level occurs.

**Fig 6:**
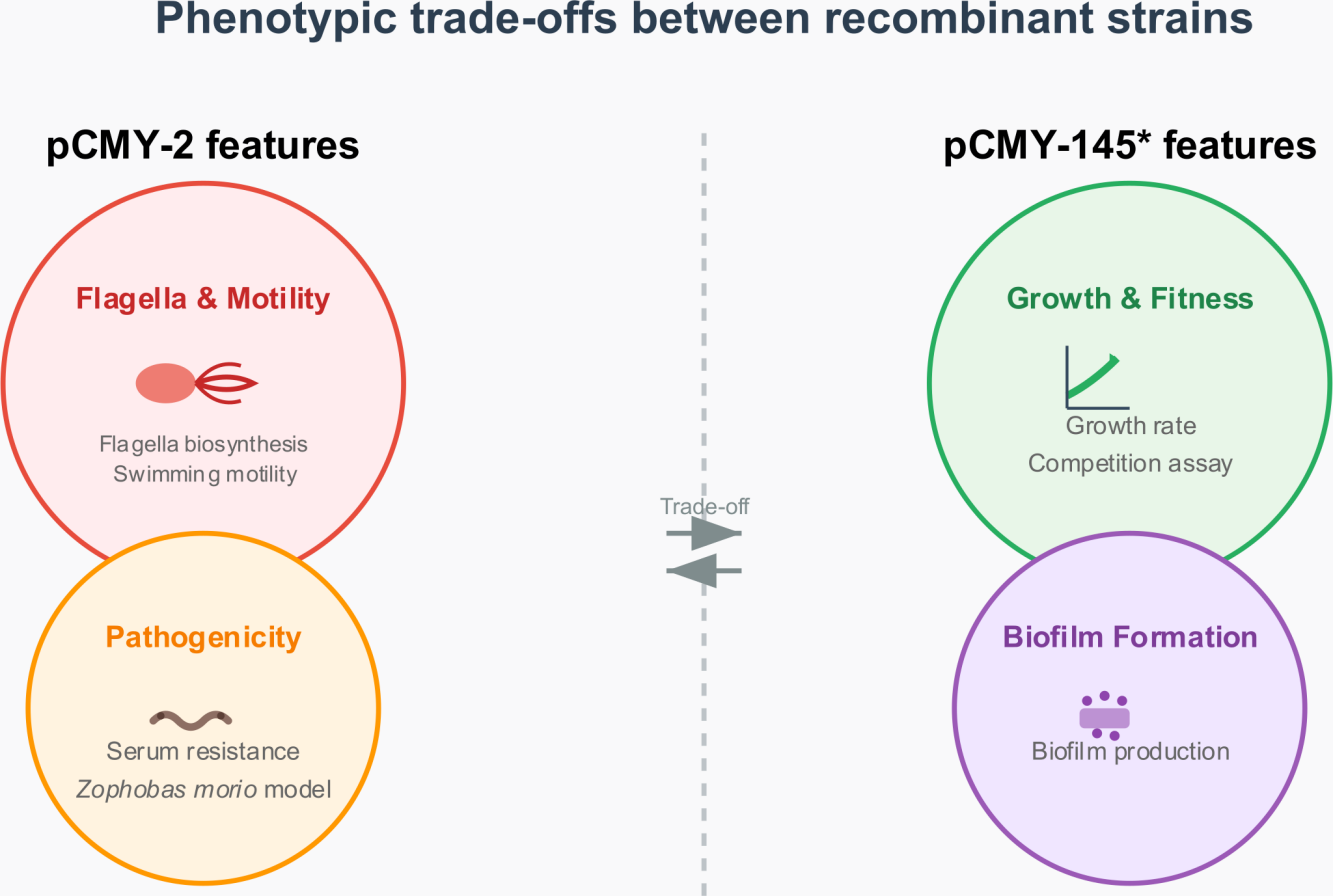
General overview of results obtained through diverse experimental approaches, comparing the pair of recombinant strains respectively producing CMY-2 and CMY-145. *Same results were obtained for pCMY-42.

In order to fully grasp the observations made through the different experimental approaches performed in the overall study, a general overview is presented in Fig. 6.

From a clinical point of view, our observations are extremely relevant, considering that CMY-2 is commonly identified among *E. coli* isolates recovered either from human infections or colonizations (44, 45). More generally, it is the most identified acquired AmpC enzyme in Enterobacterales. We might therefore consider that CMY-2, as well as DHA-1 and FOX-5, is significantly contributing to the spread and pathogenesis of the corresponding *E. coli* producers.

## MATERIALS AND METHODS

### Strain backgrounds and plasmids

Strains available in the Swiss National Reference Center for Emerging Antibiotic Resistance strain collection carrying *ampC* genes were selected for this study. This collection includes a wide range of strains producing carbapenemases, ESBLs, and AmpCs isolated from colonization and infection sites from patients hospitalized in Switzerland. Recombinant strains were constructed with WT *E. coli* strain MG1655 (K12). Recombinant plasmids were constructed using pTOPO-Blunt plasmid (Invitrogen, USA) bearing *bla*_CMY-2_, *bla*_CMY-42_, *bla*_CMY-145_, *bla*_DHA-1_, *bla*_ACC-1_, and *bla*_FOX-5_*ampC* genes and named pCMY-2, pCMY-42, pCMY-145, pDHA-1, pACC-1, and pFOX-5, respectively. In parallel, a similar-in-size recombinant plasmid named p0 consisting of pTOPO-Blunt harboring a truncated (encoding a non-functional protein) malate dehydrogenase (*mdh*) housekeeping gene of *E. coli* (same insert size) was generated. Transformation of the abovementioned different strain backgrounds was obtained by electro-transformation, and selection was based on kanamycin 50 mg/L (Sigma-Aldrich, USA). Moreover, a WT *K. pneumoniae* strain (namely CIP 53153 and referred to in the text as Kp-WT) was also used as recipient for transformation with recombinant plasmids bearing *bla*_CMY_ genes, giving rise to recombinant strains Kp-pCMY-2, Kp-pCMY-42, and Kp-pCMY-145, respectively. The p0 plasmid was also introduced in CIP 53153, giving rise to the Kp-p0 recombinant strain.

The same recombinant plasmids were also transformed into recipient *E. coli* KEIO strain (named KEIO-WT), leading to recombinant strains KEIO-p0, KEIO-pCMY-2, pCMY-42, and pCMY-145, respectively. In parallel, a KEIO strain mutant exhibiting a deleted *flhD* gene (36) was also used as recipient for transformation experiments, and corresponding recombinant strains KEIO-Δ*flhD*-p0, KEIO-*ΔflhD*-pCMY-2, KEIO-*ΔflhD*-pCMY-42, and KEIO-*ΔflhD*-pCMY-145 were obtained. Finally, site-directed mutagenesis experiments were performed using the Q5 Site-Directed Mutagenesis Kit (New England BioLabs, USA) to generate recombinant strains required for evaluating the impact of different mutations within the CMY protein sequence on the growth capacities, namely pCMY-2-Asn90Thr (or pCMY-2-N90T), pCMY-145-Ser64Arg (or pCMY-145-S64R), pDHA-1-Val211Ser (or pDHA-1-V211S), pFOX-5-V211S, and pACC-1-V211S. Primers used for cloning and performing site-directed mutagenesis are shown in Table S5.

### Measurement of growth capacities

Measurements of the respective growth rates of the different recombinant strains were performed using the different isogenic backgrounds in *E. coli* MG1655 or *K. pneumoniae* CIP 53153, following standard protocols (46, 47). Briefly, bacterial growth was measured over time at 600 nm wavelength (OD_600_) using a BioTek Cytation 5 plate reader (Agilent Technologies, CA, USA). Individual cultures were pre-cultivated overnight in Luria-Bertani (LB) broth (Bio-Rad, Cressier, Switzerland) with shaking at 37°C, with or without supplementation with kanamycin 50 mg/L. Then, cultures were normalized by measuring their OD_600_ and individually inoculated either in M9 minimal medium (M9, Avantor, USA) or in LB in a 1/50 ratio (0.1 mL of the isolate in 5 mL of M9 or LB). Culture OD_600_ was measured at 0 h and once again normalized, if necessary. After that, cultures were transferred to a 96-well Costar flat-bottom microplate (Corning Life Sciences, USA) and incubated at 37°C with continuous orbital shaking, and their OD_600_ was measured every 30 min until reaching 16 h of growth. Experiments were performed on six independent biological replicates with two technical replicates each.

### Measurement of relative bacterial fitness

In parallel to the growth curve measurements, competition assays were performed using a mix of two strains with the goal of evidencing whether one of them possessed a growth advantage regardless of any specific selective pressure. Competition assays were performed as described by Ranjan et al. (47) with some adjustments. Individual cultures were pre-cultivated in LB overnight with shaking at 37°C with or without supplementation (kanamycin 50 mg/L). Cultures were normalized by measuring their OD_600_ and inoculated in M9 in a 1/50 ratio (0.3 mL of each isolate in 15 mL of M9). Competition experiments were always performed between no more than two different isolates. Cultures were incubated at 37°C with shaking. Four time points of collection were analyzed (0 h, 4 h, 6 h, and 8 h). At each time point, 100 µL of the culture were collected and serially diluted until 10^−8^ before plating in LB agar (Carl Roth, Germany) (from 10^0^ to 10^−8^). Plates were incubated at 37°C overnight. Two dilution plates containing from 30 to 300 colony-forming units (cfu) were selected for counting and screening the isolates. Screening was performed using different methods depending upon the possibility of using antibiotic selection or not. First, competition screening was performed with antibiotic selection, where mixed cultures were plated either on kanamycin (50 mg/L) or kanamycin (50 mg/L) + ampicillin (100 mg/L) (for selection of AmpC producers) to evaluate the relative proportion of *bla*_AmpC-negative_ vs *bla*_AmpC-positive_ recombinant strains at the respective end point of the cultures.

Second, another approach was considered for measuring the relative bacterial growth during competition when there was no option for screening with antibiotic selection for two strains (e.g., pCMY-2 vs pCMY-42 or pCMY-145). Derivatives of plasmid pTOPO were constructed aiming to measure the production of the Green fluorescent protein (GFP) in its active or non-active form, by constructing recombinant pTOPO plasmids harboring the corresponding GFP-encoding gene on one hand, and then by inserting a stop codon in the initial part of the GFP using site-directed mutagenesis (primers used are shown in Table S5). Then, mixed cultures were plated on kanamycin plates (50 mg/L). For both strategies, the calculation and interpretation of the relative fitness (*w*) were performed according to the method described by Elsener et al. (48). Experiments were performed in three independent biological replicates with two technical replicates each.

### Motility assays

Swimming motility assays were performed in 0.3% LB agar plates. Bacterial isolates were pre-cultivated in LB agar, with or without 50 mg/L kanamycin, at 37°C overnight. Single colonies were then inoculated at the center of the soft LB agar plates. The diameters of the motility halos were measured at 18 h, 24 h, 48 h, and 72 h of incubation at 37°C, without any agitation. Each experiment was conducted in six independent biological replicates. Imaging of the swimming plates was performed using a Bio-Rad Gel Doc XR imaging system (Bio-Rad, USA).

### Biofilm formation assays

Biofilm assays were performed as previously reported (49), with adaptations. Briefly, overnight cultures of *E. coli* in LB broth were normalized, and 100 μL of each strain was inoculated in a 96-well Costar flat-bottom microplate for 24 h at 37°C without agitation. After incubation, cells were dumped out of the microplate, and the microplates were submerged in distilled water. This process was repeated to reduce background staining. Then, 125 μL of a 0.1% solution of crystal violet was added to each well and incubated at room temperature (RT) for 15 min. After that, microplates were rinsed in distilled water by submersion three to four times and left to dry for a few hours or overnight. Then, 125 µL of 30% acetic acid was added to each well to solubilize the stained biofilm, and the microplates were incubated at RT for 15 min. The volumes were transferred to a new 96-well microplate and absorbances were quantified in a SpectraMax iD5 (Molecular Devices LLC) plate reader at 550 nm. Absorbances were normalized using the negative control (medium without bacteria), and the production of biofilm was achieved by comparing the absorbance of the tested strain with the positive control (*E. coli* K12). Experiments were performed in three biological replicates with eight technical replicates each.

### Serum resistance assays

Bacteria were pre-cultivated overnight without shaking in LB or LB plus 50 mg/L kanamycin at 37°C. Then, they were inoculated in a fresh LB or LB plus 50 mg/L kanamycin and incubated without shaking at 37°C until reaching an OD_600_ of 0.5–0.6. After that, cells were centrifuged, and the pellet was resuspended in 10 mM PBS (Sigma-Aldrich, USA). Cells were washed once again with PBS. After that, resuspended cells were inoculated in 33% human serum (Sigma-Aldrich, USA) and 33% inactivated human serum (Sigma-Aldrich, USA) at a 1:2 ratio, respectively, and incubated at 37°C for 3 h. Both suspensions (with human serum and with inactivated human serum) were serially diluted until 10^−8^ before plating on LB agar (from 10^0^ to 10^−8^) and incubated at 37°C overnight. Two dilution plates of each condition (with human serum and with inactivated human serum) containing from 30 to 300 CFU were selected for counting the isolates. Calculations were performed dividing the number of CFU/mL obtained in human serum by the number of CFU/mL obtained in inactivated human serum. Experiments were performed in three independent replicates.

### Measurement of bacterial growth/pathogenicity using an *in vivo* model

An invertebrate infection model using *Zophobas morio* larvae (*Zm*L) was employed to assess the relative pathogenicity of the different strains and mutants *in vivo*. This system has been well established for assessing the ability of given strains to colonize, replicate, and kill those larvae and was useful to evaluate the impact of given mutations on larval pathogenicity (50, 51). Late-instar *Zm*L, derived from BUGS-International GmbH stocks and obtained via a Swiss pet store, was laboratory-reared as described previously (52). Three independent sets of 10 larvae per challenge inoculum were tested. Briefly, larvae were temporarily immobilized by placing them in a petri dish on ice for 10 min prior to injection. Challenge inocula, 10^7^ CFU/larva of each strain or 1× Dulbecco’s phosphate-buffered saline (dPBS), were administered in 15 μL volume through the second ventral segment using a 26-gauge beveled needle (30°C oriented upward) and a 250 μL Hamilton Gastight 1700 syringe. The needle was immersed in 70% ethanol between injections, and the syringe was filled with 70% ethanol for 3 min and subsequently rinsed five times each with 70% ethanol and dPBS between strain procedures, as previously described (53). After the challenge, larvae were placed in a petri dish and kept at RT with daily slices of pear for 5 days. Individual *Zm*L (*n* = 30) was observed at random throughout each experimental condition (*n* = 9) at five time points post-infection (24 h, 48 h, 72 h, 96 h, 120 h). An established health index scoring system for *Galleria mellonella* larvae was adapted from Loh et al. (54) to reflect the unique physiological characteristics of *Zm*L (activity, molting, melanization, and survival). Each *Zm*L was assessed across attributes, with scores combined to generate a composite health index ranging from 0 (infected, dead larva) to 9–10 (healthy, uninfected larva). Detailed attribute scoring is summarized in Table S4.

### Global transcriptomic analysis by RNA sequencing

In order to identify which genes could be involved in the phenotypic observations, transcriptomic assays measuring the up- or downregulation of genes were performed. The methodology used was the following: for each strain, RNA extraction was performed in three biological replicates using the Zymo kit (Zymo Research, USA) on 10 mL of bacterial cultures that had reached an OD_600_ of 0.6. DNase treatment was carried out using the Turbo DNase kit (Invitrogen, USA). After quality checking with a TapeStation (Agilent Technologies, USA), RNA samples were sent to GeneWiz (Azenta Life Sciences, Germany) for rRNA depletion, followed by cDNA library preparation and strand-specific RNA sequencing. Raw data were paired, trimmed, and mapped against the reference genome of *E. coli* K12 using the CLC software (Qiagen, USA). Downstream analyses, including principal component analysis, unsupervised heat map, and differential expression analysis (DE), were also performed. A cutoff of two log_2_ fold changes and FDR < 0.05 was chosen to narrow down the deregulated transcripts. To perform GO enrichment, we used the Panther tool version 19.0 (https://pantherdb.org/) and the STRING tool version 12.0 (https://string-db.org/). STRING was also used to generate a STRING network with FDR < 0.05.

### RT-qPCR

Since preliminary transcriptomic assays showed that the expression of the *flhD* and *flhC* genes was significantly downregulated upon production of CMY-42 or CMY-145, a focus was made on selected genes to determine their level of expression. To avoid performing systematic whole-transcriptome analysis for all generated recombinant strains, including those with different clinically relevant genetic background as explained previously, quantitative RT-qPCR was used. Using RT-qPCR, we measured specifically the respective expression level of those different genes encompassed in the so-called flagellar genes operon, namely *flhC*, *fliA*, and *flgK*. Other genes not related to the flagellar biosynthesis cascade and that were upregulated upon production of CMY-42 or CMY-145 (i.e., *gspC*, encoding a type II secretion system protein, and *dsdA*, encoding a D-serine ammonia-lyase) were also analyzed. Primers used for the RT-qPCR are listed in Table S5.

mRNA samples were obtained after reaching a bacterial OD_600_ of 0.5–0.6. Growth was performed with kanamycin (50 mg/L) pressure in minimal M9. Isolation of total RNA was performed using the Quick-RNA Miniprep kit (Zymo Research, USA). RNA samples were subsequently treated with a Turbo DNA-free kit. RNA concentration and purity were measured using the Nanodrop 2000 Spectrophotometer (Thermo Scientific, Switzerland). Finally, cDNA synthesis was performed with the LunaScript RT SuperMix kit (New England BioLabs, USA).

### Protein purification

The different AmpC variants, namely, CMY-2, CMY-42, and CMY-145, were cloned into pOPINF expression plasmid using the In-Fusion cloning kit (Takara Bio, Japan) and giving rise to pOPINF+CMY-2, pOPINF+CMY-42, and pOPINF+CMY-145 (55). Primers are listed in Table S5. Constructed plasmids were transformed into *E. coli* BL21 DE3 (pLysS) strain. Cultures were inoculated in LB medium supplemented with 100 mg/L ampicillin at 37°C until OD_600_ reached 0.8, then induced with 0.5 mM/L isopropyl β-D-1-thiogalactopyranoside (Carl Roth, Germany) overnight at 16°C. Cells were harvested, resuspended in lysozyme (2 mg/L), and incubated at RT for 20 min. Then cells were sonicated using an ultrasonic processor Vibra-Cell (Avantor, USA). Lysates were centrifuged at 11,000 rpm for 10 min at 4°C, and the soluble fraction was filter-sterilized (0.22 µm). The lysates were added to a nickel-nitrilotriacetic acid (Ni-NTA) agarose column (His GraviTrap GE Healthcare, USA). The column was washed with a wash buffer (100 mM/L Tris-HCl, 300 mM/L NaCl, 20 mM/L imidazole [pH 8.0], and 10% glycerol) and eluted with an elution buffer (100 mM/L Tris-HCl, 300 mM/L NaCl, 400 mM/L imidazole [pH 8.0], and 10% glycerol). Purified proteins were analyzed by SDS-PAGE.

### EMSA

Purified proteins (CMY-2, CMY-42, and CMY-145) were used to perform the experiments using the EMSA kit (Invitrogen, USA). The amplified *flhD* gene, including or not its native promoter (see primers shown in Table S5), hence two distinct amplicons, was used as targets for assays aimed at evidencing potential binding between CMY enzymes and the flagellar biosynthesis master regulator FlhD_4_C_2_. DNA fragments were incubated at a fixed concentration of 40 ng together with the different purified CMY proteins in increasing concentrations (from 65 ng to 1,040 ng) with the binding buffer provided for 20 min, following manufacturer’s instructions. Mixtures were supplemented with a loading buffer and inoculated in an mPAGE 4-12% Bis-Tris gel (Merck, Germany) in 1× MOPS buffer. The gels were stained with SYBR provided from the mentioned kit and visualized under UV light (254–300 nm).

### Transmission electron microscopy

Firstly, the formvar/carbon-coated grids (Electron Microscopy Sciences, USA) were glow discharged at 2 × 10⁻¹ mbar and 2 mA for 8 s to improve bacterial cell attachment. This process was performed using an ELMO glow discharge system equipment (Cordouan Technologies, France). After overnight pre-cultivation in LB or LB plus 50 mg/L kanamycin, isolates were normalized to an OD_600_ 0.5–0.6. Then, a drop (30 µL) of the bacterial cultures was spotted on parafilm, and one grid was floated on top of the drop for 7 min. After that, the grid was transferred to a 0.22 µm-filtered Milli-Q water drop and incubated for 15 s. This step was repeated three times. The grids were dried on filter paper and were ready to be observed under a Tecnai G2 Spirit BioTwin (FEI Company, USA) transmission electron microscope at 120 kV equipped with a WA Veleta camera (Olympus, Japan).

### Statistical analyses and visualization tools

Statistically significant differences were assessed by performing multiple methods. Two-tailed parametric *t*-tests and one-way ANOVA followed by Tukey’s multiple comparisons statistical test were performed using GraphPad Prism version 9.5.1. Plate images of competition assays were documented using the Genesys G: Box Chemi XRQ (Syngene, India). The Venn diagrams were generated using the Venn draw tool from Bioinformatics & Evolutionary Genomics website (https://bioinformatics.psb.ugent.be/webtools/Venn/). Volcano plots were generated using the MaGIC Volcano Plot Tool (https://volcano.bioinformagic.tools/). The *in vivo* model health score based on treatment and time point was conducted on R v.4.4.2 by ordinal logistic regression followed by a *post hoc* analysis (estimated marginal means) using MASS v.7.3-65 and emmeans v1.11.1 packages, respectively. STRING tool version 12.0 was used to generate a STRING network with a highest confidence score of 0.900 and FDR < 0.05 corrected by Benjamini-Hochberg (https://string-db.org/). All graphs were constructed using GraphPad Prism version 9.5.1. Statistically significant results were defined with a confidence level of 95% (*P* < 0.05).

## Data Availability

The raw and processed data generated in this study have been submitted to the Gene Expression Omnibus (GEO) repository at the National Center for Biotechnology Information (NCBI) under the accession number GSE311194.

## SUPPLEMENTAL MATERIAL

Supplemental material mbio.00088-26-s0001.docx Supplemental figures and tables.

Table S3 mbio.00088-26-s0002.xlsx Raw RNA sequence data.

ASM does not own the copyrights to Supplemental Material that may be linked to, or accessed through, an article. The authors have granted ASM a non-exclusive, world-wide license to publish the Supplemental Material files. Please contact the corresponding author directly for reuse.

## References

[1] Bush K. 2018. Past and present perspectives on β-lactamases. Antimicrob Agents Chemother 62:e01076-18. doi:10.1128/AAC.01076-18 30061284 PMC6153792

[2] Bush K. 2013. The ABCD’s of β-lactamase nomenclature. J Infect Chemother 19:549–559. doi:10.1007/s10156-013-0640-7 23828655

[3] Ambler RP. 1980. The structure of β-lactamases. Philos Trans R Soc Lond B Biol Sci 289:321–331. doi:10.1098/rstb.1980.0049 6109327

[4] Philippon A, Jacquier H, Ruppé E, Labia R. 2019. Structure-based classification of class A beta-lactamases, an update. Curr Res Transl Med 67:115–122. doi:10.1016/j.retram.2019.05.003 31155436

[5] Livermore DM, Canton R, Gniadkowski M, Nordmann P, Rossolini GM, Arlet G, Ayala J, Coque TM, Kern-Zdanowicz I, Luzzaro F, Poirel L, Woodford N. 2007. CTX-M: changing the face of ESBLs in Europe. J Antimicrob Chemother 59:165–174. doi:10.1093/jac/dkl483 17158117

[6] Bevan ER, Jones AM, Hawkey PM. 2017. Global epidemiology of CTX-M β-lactamases: temporal and geographical shifts in genotype. J Antimicrob Chemother 72:2145–2155. doi:10.1093/jac/dkx146 28541467

[7] Philippon A, Arlet G, Labia R, Iorga BI. 2022. Class C β-lactamases: molecular characteristics. Clin Microbiol Rev 35:e00150-21. doi:10.1128/cmr.00150-21 35435729 PMC9491196

[8] Juan C, Torrens G, González-Nicolau M, Oliver A. 2017. Diversity and regulation of intrinsic β-lactamases from non-fermenting and other Gram-negative opportunistic pathogens. FEMS Microbiol Rev 41:781–815. doi:10.1093/femsre/fux043 29029112

[9] Jacoby GA. 2009. AmpC β-Lactamases. Clin Microbiol Rev 22:161–182. doi:10.1128/CMR.00036-08 19136439 PMC2620637

[10] Girlich D, Naas T, Bellais S, Poirel L, Karim A, Nordmann P. 2000. Biochemical-genetic characterization and regulation of expression of an ACC-1-like chromosome-borne cephalosporinase from Hafnia alvei. Antimicrob Agents Chemother 44:1470–1478. doi:10.1128/AAC.44.6.1470-1478.2000 10817695 PMC89899

[11] Poirel L, Guibert M, Girlich D, Naas T, Nordmann P. 1999. Cloning, sequence analyses, expression, and distribution of ampC-ampR from Morganella morganii clinical isolates. Antimicrob Agents Chemother 43:769–776. doi:10.1128/AAC.43.4.769 10103179 PMC89205

[12] Barlow M, Hall BG. 2002. Origin and evolution of the AmpC β-lactamases of Citrobacter freundii. Antimicrob Agents Chemother 46:1190–1198. doi:10.1128/AAC.46.5.1190-1198.2002 11959544 PMC127158

[13] Ebmeyer S, Kristiansson E, Larsson DGJ. 2019. The mobile FOX AmpC beta-lactamases originated in Aeromonas allosaccharophila. Int J Antimicrob Agents 54:798–802. doi:10.1016/j.ijantimicag.2019.09.017 31600552

[14] Knox JR, Moews PC, Frere J-M. 1996. Molecular evolution of bacterial β-lactam resistance. Chem Biol 3:937–947. doi:10.1016/S1074-5521(96)90182-9 8939710

[15] Massova I, Mobashery S. 1998. Kinship and diversification of bacterial penicillin-binding proteins and β-lactamases. Antimicrob Agents Chemother 42:1–17. doi:10.1128/AAC.42.1.1 9449253 PMC105448

[16] Massova I, Mobashery S. 1999. Structural and mechanistic aspects of evolution of beta-lactamases and penicillin-binding proteins. Curr Pharm Des 5:929–937. 10539997

[17] Barceló IM, Jordana-Lluch E, Escobar-Salom M, Torrens G, Fraile-Ribot PA, Cabot G, Mulet X, Zamorano L, Juan C, Oliver A. 2022. Role of enzymatic activity in the biological cost associated with the production of AmpC β-lactamases in Pseudomonas aeruginosa. Microbiol Spectr 10:e02700-22. doi:10.1128/spectrum.02700-22 36214681 PMC9604156

[18] Morosini MI, Ayala JA, Baquero F, Martínez JL, Blázquez J. 2000. Biological cost of AmpC production for Salmonella enterica serotype Typhimurium. Antimicrob Agents Chemother 44:3137–3143. doi:10.1128/AAC.44.11.3137-3143.2000 11036037 PMC101617

[19] Colquhoun JM, Farokhyfar M, Anderson AC, Bethel CR, Bonomo RA, Clarke AJ, Rather PN. 2023. Collateral changes in cell physiology associated with ADC-7 β-lactamase expression in Acinetobacter baumannii. Microbiol Spectr 11:e04646-22. doi:10.1128/spectrum.04646-22 37074187 PMC10269689

[20] Carroll AC, Wong A. 2018. Plasmid persistence: costs, benefits, and the plasmid paradox. Can J Microbiol 64:293–304. doi:10.1139/cjm-2017-0609 29562144

[21] San Millan A, MacLean RC. 2017. Fitness costs of plasmids: a limit to plasmid transmission. Microbiol Spectr 5. doi:10.1128/microbiolspec.mtbp-0016-2017 PMC11687550 28944751

[22] Billane K, Harrison E, Cameron D, Brockhurst MA. 2022. Why do plasmids manipulate the expression of bacterial phenotypes? Philos Trans R Soc Lond B Biol Sci 377:20200461. doi:10.1098/rstb.2020.0461 34839708 PMC8628079

[23] Baltrus DA. 2013. Exploring the costs of horizontal gene transfer. Trends Ecol Evol 28:489–495. doi:10.1016/j.tree.2013.04.002 23706556

[24] Ahmad M, Prensky H, Balestrieri J, ElNaggar S, Gomez-Simmonds A, Uhlemann AC, Traxler B, Singh A, Lopatkin AJ. 2023. Tradeoff between lag time and growth rate drives the plasmid acquisition cost. Nat Commun 14:2343. doi:10.1038/s41467-023-38022-6 37095096 PMC10126158

[25] Sadek M, Poirel L, Nordmann P. 2021. Occurrence of aztreonam-avibactam-resistant NDM-5-producing Escherichia coli in the food chain. Antimicrob Agents Chemother 65:e00882-21. doi:10.1128/AAC.00882-21 34125588 PMC8370230

[26] Tellapragada C, Razavi M, Peris PS, Jonsson P, Vondracek M, Giske CG. 2024. Resistance to aztreonam-avibactam among clinical isolates of Escherichia coli is primarily mediated by altered penicillin-binding protein 3 and impermeability. Int J Antimicrob Agents 64:107256. doi:10.1016/j.ijantimicag.2024.107256 38925228

[27] Haidar G, Kline EG, Kitsios GD, Wang X, Kwak EJ, Newbrough A, Friday K, Hughes Kramer K, Shields RK. 2024. Emergence of high-level aztreonam-avibactam and cefiderocol resistance following treatment of an NDM-producing Escherichia coli bloodstream isolate exhibiting reduced susceptibility to both agents at baseline. JAC Antimicrob Resist 6:dlae141. doi:10.1093/jacamr/dlae141 39239090 PMC11375572

[28] Mack AR, Barnes MD, Taracila MA, Hujer AM, Hujer KM, Cabot G, Feldgarden M, Haft DH, Klimke W, van den Akker F, et al.. 2020. A standard numbering scheme for class C β-lactamases. Antimicrob Agents Chemother 64:e01841-19. doi:10.1128/AAC.01841-19 31712217 PMC7038296

[29] Aldridge P, Hughes KT. 2002. Regulation of flagellar assembly. Curr Opin Microbiol 5:160–165. doi:10.1016/s1369-5274(02)00302-8 11934612

[30] Chevance FFV, Hughes KT. 2008. Coordinating assembly of a bacterial macromolecular machine. Nat Rev Microbiol 6:455–465. doi:10.1038/nrmicro1887 18483484 PMC5963726

[31] Minamino T, Imada K. 2015. The bacterial flagellar motor and its structural diversity. Trends Microbiol 23:267–274. doi:10.1016/j.tim.2014.12.011 25613993

[32] Sun H, Wang M, Liu Y, Wu P, Yao T, Yang W, Yang Q, Yan J, Yang B. 2022. Regulation of flagellar motility and biosynthesis in enterohemorrhagic Escherichia coli O157:H7. Gut Microbes 14:2110822. doi:10.1080/19490976.2022.2110822 35971812 PMC9387321

[33] Sun G, Yu Z, Li Q, Zhang Y, Wang M, Liu Y, Liu J, Liu L, Yu X. 2023. Mechanism of Escherichia coli lethality caused by overexpression of flhDC, the flagellar master regulator genes, as revealed by transcriptome analysis. Int J Mol Sci 24:14058. doi:10.3390/ijms241814058 37762361 PMC10530849

[34] Khan F, Tabassum N, Pham DTN, Oloketuyi SF, Kim YM. 2020. Molecules involved in motility regulation in Escherichia coli cells: a review. Biofouling 36:889–908. doi:10.1080/08927014.2020.1826939 33028083

[35] Osterman IA, Dikhtyar YY, Bogdanov AA, Dontsova OA, Sergiev PV. 2015. Regulation of flagellar gene expression in bacteria. Biochemistry (Moscow) 80:1447–1456. doi:10.1134/S000629791511005X 26615435

[36] Baba T, Ara T, Hasegawa M, Takai Y, Okumura Y, Baba M, Datsenko KA, Tomita M, Wanner BL, Mori H. 2006. Construction of Escherichia coli K-12 in-frame, single-gene knockout mutants: the Keio collection. Mol Syst Biol 2:2006. doi:10.1038/msb4100050 PMC1681482 16738554

[37] Yamanaka Y, Aizawa SI, Yamamoto K. 2022. The hdeD gene represses the expression of flagellum biosynthesis via LrhA in Escherichia coli K-12. J Bacteriol 204:e0042021. doi:10.1128/JB.00420-21 34694904 PMC8765402

[38] Macnab RM. 1996. *Escherichia coli* and *Salmonella*: cellular and molecular biology, p 123–145. In Neidhardt FC (ed), Flagella and motility, 2nd ed. ASM Press, Washington, DC.

[39] Plague GR, Boodram KS, Dougherty KM, Bregg S, Gilbert DP, Bakshi H, Costa D. 2017. Transposable elements mediate adaptive debilitation of Flagella in experimental Escherichia coli populations. J Mol Evol 84:279–284. doi:10.1007/s00239-017-9797-5 28646326 PMC6002858

[40] Ni B, Ghosh B, Paldy FS, Colin R, Heimerl T, Sourjik V. 2017. Evolutionary remodeling of bacterial motility checkpoint control. Cell Rep 18:866–877. doi:10.1016/j.celrep.2016.12.088 28122238 PMC5289928

[41] Ni B, Colin R, Link H, Endres RG, Sourjik V. 2020. Growth-rate dependent resource investment in bacterial motile behavior quantitatively follows potential benefit of chemotaxis. Proc Natl Acad Sci USA 117:595–601. doi:10.1073/pnas.1910849117 31871173 PMC6955288

[42] Sidjabat HE, Paterson DL, Qureshi ZA, Adams-Haduch JM, O’Keefe A, Pascual A, Rodríguez-Baño J, Doi Y. 2009. Clinical features and molecular epidemiology of CMY-type β-lactamase–producing Escherichia coli. Clin Infect Dis 48:739–744. doi:10.1086/597037 19187027 PMC2711437

[43] Winokur PL, Vonstein DL, Hoffman LJ, Uhlenhopp EK, Doern GV. 2001. Evidence for transfer of CMY-2 AmpC β-lactamase plasmids between Escherichia coli and Salmonella isolates from food animals and humans. Antimicrob Agents Chemother 45:2716–2722. doi:10.1128/AAC.45.10.2716-2722.2001 11557460 PMC90722

[44] Koga VL, Maluta RP, da Silveira WD, Ribeiro RA, Hungria M, Vespero EC, Nakazato G, Kobayashi RKT. 2019. Characterization of CMY-2-type beta-lactamase-producing Escherichia coli isolated from chicken carcasses and human infection in a city of south Brazil. BMC Microbiol 19:174. doi:10.1186/s12866-019-1550-3 31362706 PMC6664532

[45] Pietsch M, Irrgang A, Roschanski N, Brenner Michael G, Hamprecht A, Rieber H, Käsbohrer A, Schwarz S, Rösler U, Kreienbrock L, Pfeifer Y, Fuchs S, Werner G, RESET Study Group. 2018. Whole genome analyses of CMY-2-producing Escherichia coli isolates from humans, animals and food in Germany. BMC Genomics 19:601. doi:10.1186/s12864-018-4976-3 30092762 PMC6085623

[46] Reddy CA. 2007. Methods for general and molecular microbiology. 3rd ed. ASM Press, Washington, D.C.

[47] Ranjan A, Scholz J, Semmler T, Wieler LH, Ewers C, Müller S, Pickard DJ, Schierack P, Tedin K, Ahmed N, Schaufler K, Guenther S. 2018. ESBL-plasmid carriage in E. coli enhances in vitro bacterial competition fitness and serum resistance in some strains of pandemic sequence types without overall fitness cost. Gut Pathog 10:24. doi:10.1186/s13099-018-0243-z 29983750 PMC6003029

[48] Elsener TA, Cehovin A, Philp C, Fortney K, Spinola SM, Maiden MCJ, Tang CM. 2025. Origin, evolution, and success of pbla, the gonococcal beta-lactamase plasmid, and implications for public health. PLoS Pathog 21:e1013151. doi:10.1371/journal.ppat.1013151 40327678 PMC12080925

[49] O’Toole GA. 2011. Microtiter dish biofilm formation assay. J Vis Exp 47:2437. doi:10.3791/2437 PMC3182663 21307833

[50] Rakovitsky N, Temkin E, Hameir A, Lurie-Weinberger M, Keren-Paz A, Carmeli Y. 2024. Zophobas morio larvae as a novel model for the study of Acinetobacter virulence and antimicrobial resistance. Front Microbiol 15:1375787. doi:10.3389/fmicb.2024.1375787 38476953 PMC10927975

[51] Rakovitsky N, Lurie-Weinberger M, Keren-Paz A, Carmeli Y. 2025. Genomic and phenotypic characterization of six multidrug-resistant Acinetobacter pittii isolates. Sci Rep 15:29577. doi:10.1038/s41598-025-15848-2 40797094 PMC12344141

[52] Aldeia C, Campos-Madueno EI, Endimiani A. 2025. A commercial bacteriophage cocktail failed to decolonize Zophobas morio larvae and promoted overgrowth of an OXA-48-producing Salmonella enterica. Eur J Clin Microbiol Infect Dis. doi:10.1007/s10096-025-05275-6 PMC12987905 41131385

[53] Russo TA, MacDonald U. 2020. The Galleria mellonella infection model does not accurately differentiate between hypervirulent and classical Klebsiella pneumoniae. mSphere 5:e00850-19. doi:10.1128/mSphere.00850-19 31915230 PMC6952204

[54] Loh JMS, Adenwalla N, Wiles S, Proft T. 2013. Galleria mellonella larvae as an infection model for group A streptococcus. Virulence 4:419–428. doi:10.4161/viru.24930 23652836 PMC3714134

[55] Berrow NS, Alderton D, Sainsbury S, Nettleship J, Assenberg R, Rahman N, Stuart DI, Owens RJ. 2007. A versatile ligation-independent cloning method suitable for high-throughput expression screening applications. Nucleic Acids Res 35:e45. doi:10.1093/nar/gkm047 17317681 PMC1874605

