# Supplementary material for: Beneficial impact of acquired AmpC β-lactamases on bacterial fitness and pathogenicity: a new paradigm
Source: mBio. 2026 Apr 13;17(5):e00088-26. doi: 10.1128/mbio.00088-26 (PMC13170162; doi:10.1128/mbio.00088-26)
Supplement: Supplemental material — Supplemental figures and tables. [file mbio.00088-26-s0001.docx]

**Supplementary Materials**


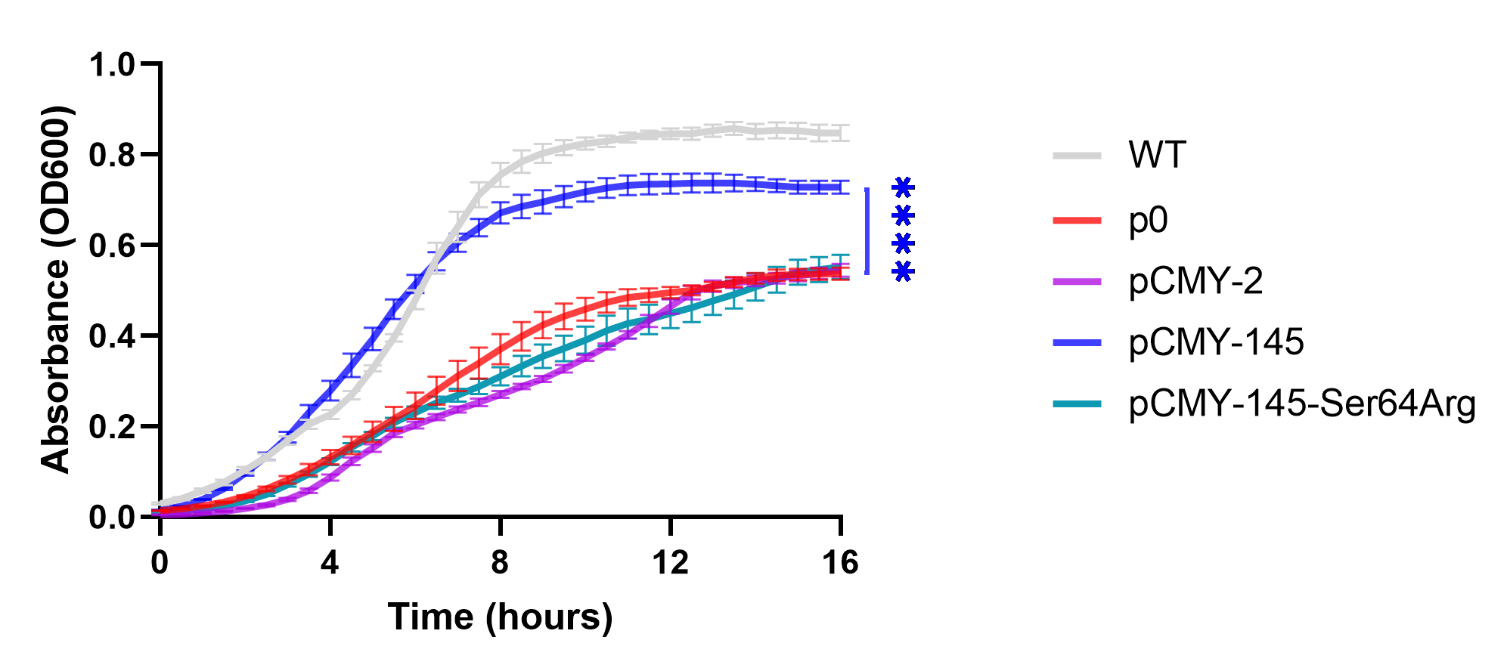


**Figure S1**. Measurement of growth capacities of pCMY-145-Ser64Arg compared with pCMY-145. WT, p0, and pCMY-2 are shown as controls of the experiment. Data is shown as mean ± standard error of mean (SEM). ****, P-value ≤ 0.0001.

**
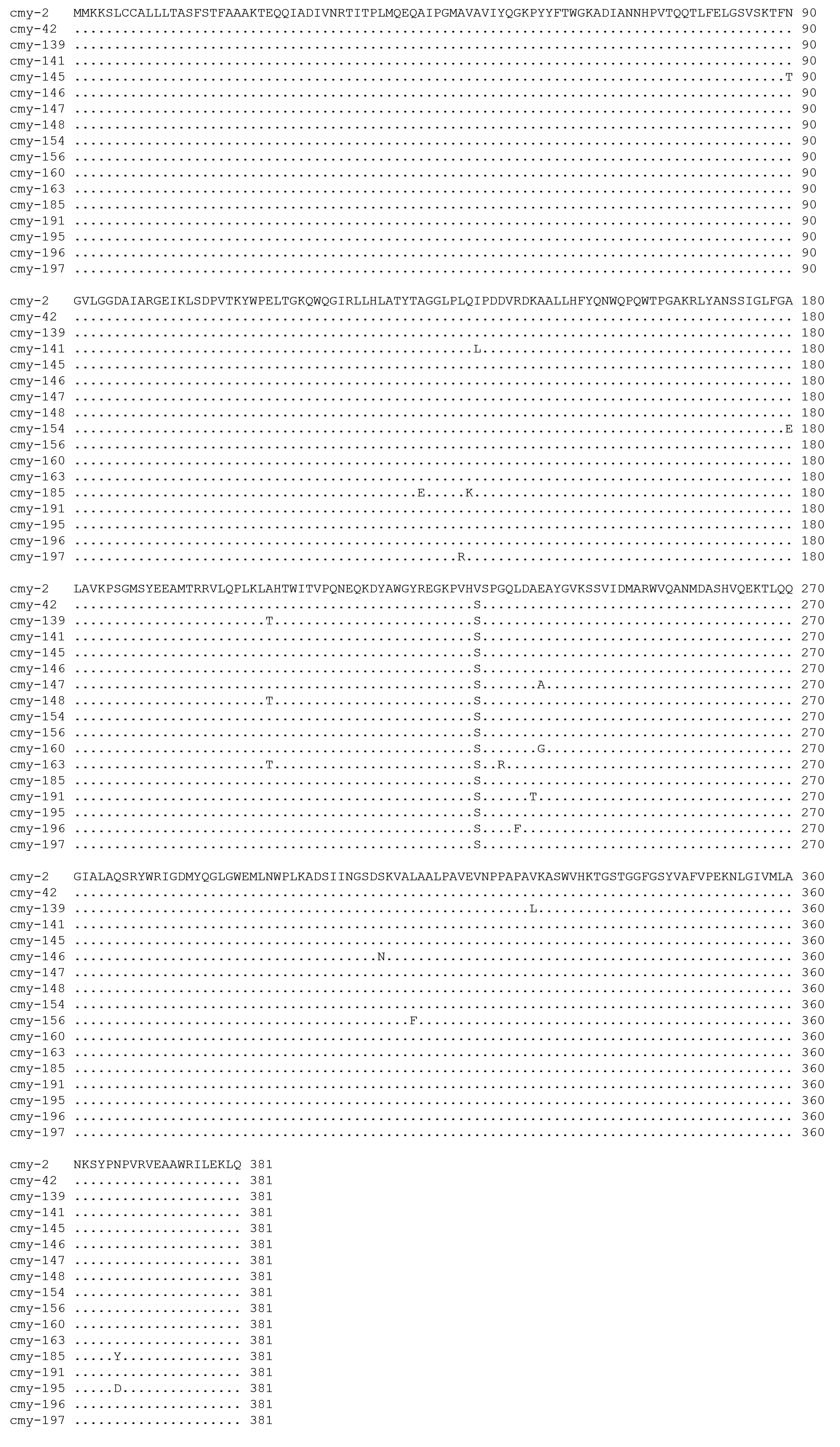
**

**Figure S2**. Multiple sequence alignment of CMY-2 and CMY variants with the Ser211 mutation. Nomenclature is based on the structural alignment-based numbering of class C β-lactamase (SANC). (Mack et al 2020.^24^ (-) identical residue compared with the CMY-2 sequence.

**

**

**Figure S3. Demonstration of competition assay between pCMY-2 carrying GFP protein with a stop codon inserted (pCMY-2-GFP-stop) and pCMY-145 carrying the GFP protein active (pCMY-145-GFP). Blue colonies represent pCMY-2 and green fluorescent colonies pCMY-145. Images were documented using the Genesys G: Box Chemi XRQ (Syngene, India). Representative figure that can be extrapolated to pCMY-42 results when comparing with pCMY-2.**


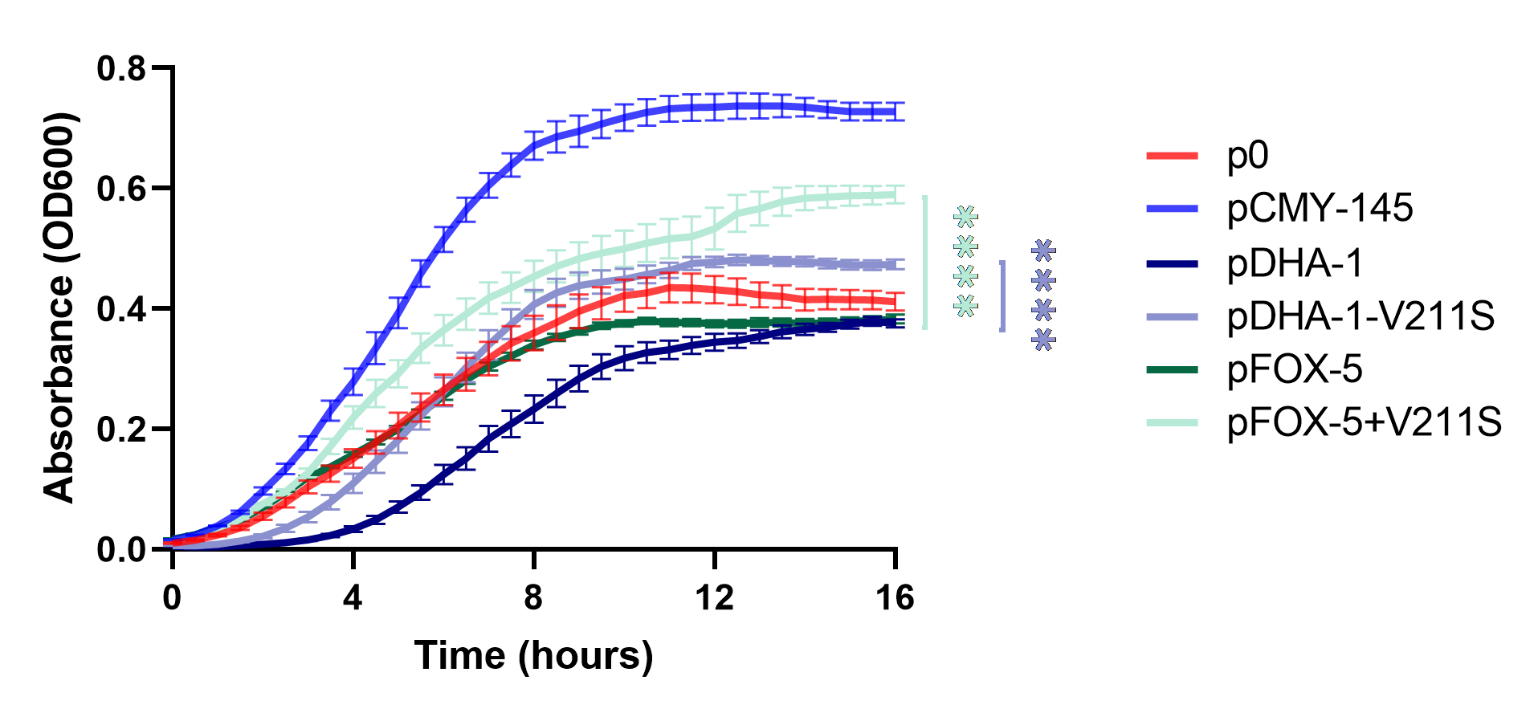


**Figure S4**. Measurement of growth capacities of pDHA-1 and pFOX-5 against their single amino-acid variant constructed pDHA-1-V211S and pFOX-5-V211S. p0 and pCMY-145 are shown as controls of the experiment. Data is shown as mean ± SEM. ****, P-value ≤ 0.0001.

**
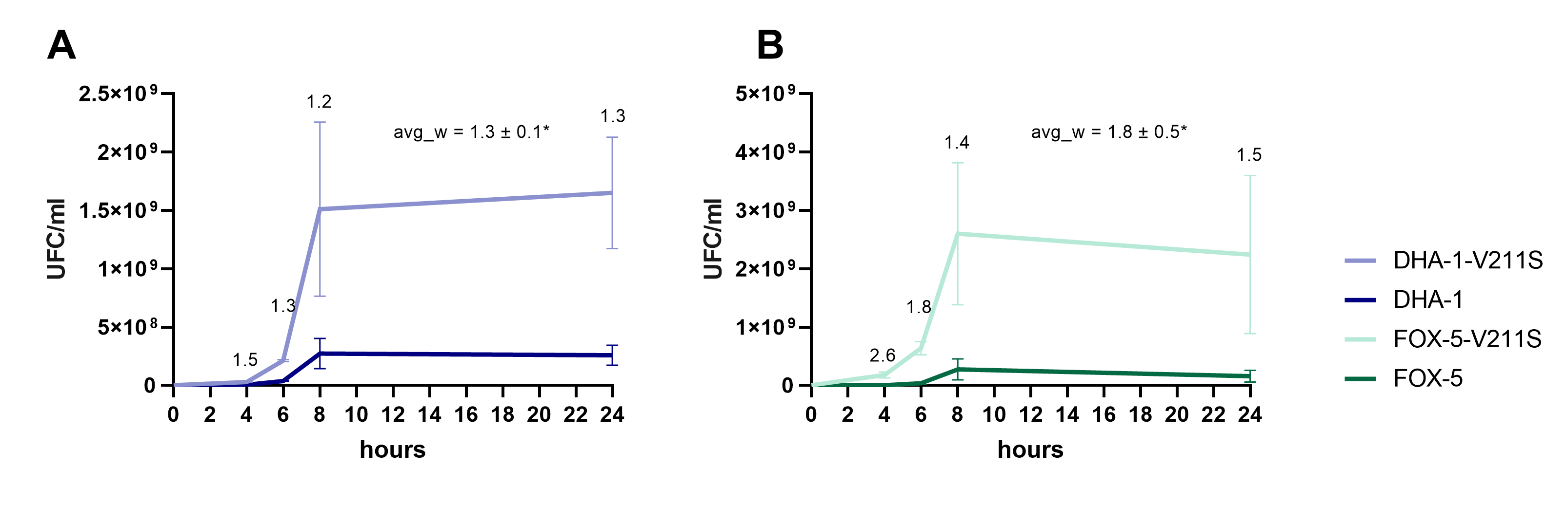
**

**Figure S5.** Competition assays. **A.** Competition assay showing relative fitness advantage for pDHA-1-V211S over pDHA-1. **B.** Competition assay showing relative fitness advantage for pFOX-5-V211S over pFOX-5. Data is shown as mean ± SEM. Numbers show relative fitness in each timepoint (4h, 6h, 8h, and 24h); Avg_w shows global average relative fitness ± SEM: *, P-value ≤ 0.05.


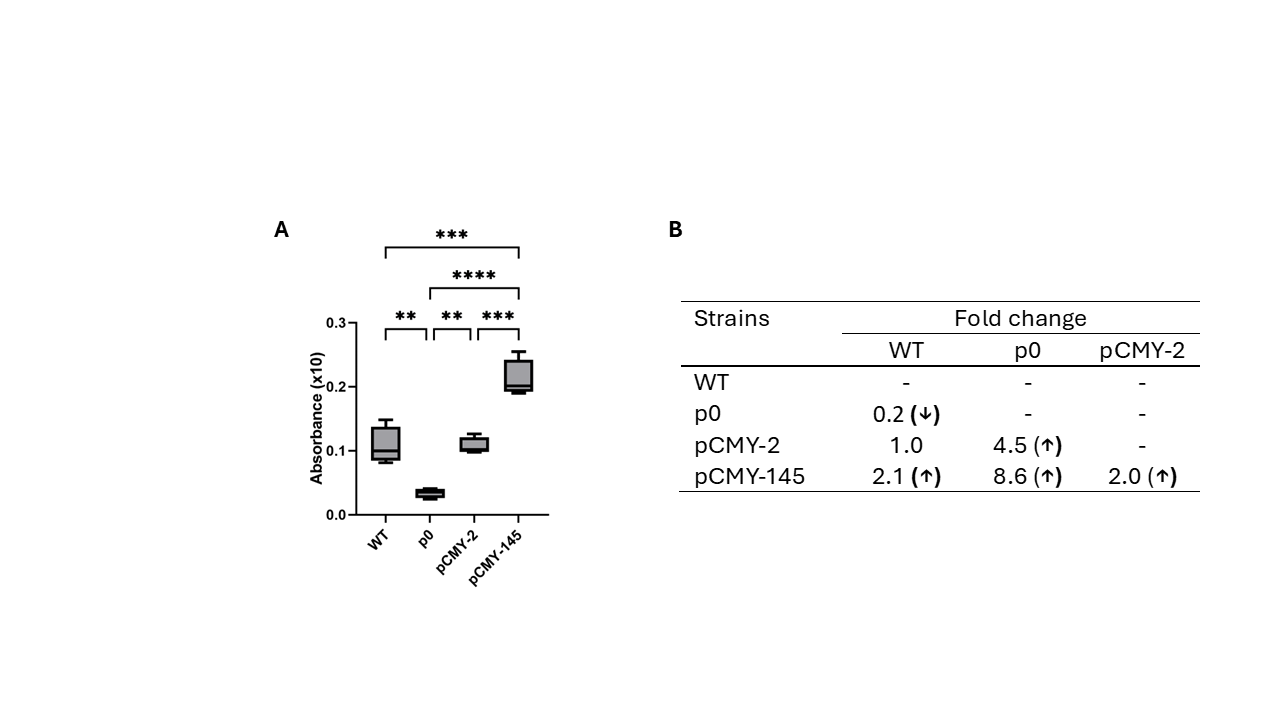


**Figure S6**. Biofilm production for recombinant isogenic strains and wild type (WT). **A.** Box & whiskers chart showing absorbance measures. Data is shown as box and whisker plots indicating the median (line), interquartile range (box), and minimum and maximum values (whiskers). *, P-value ≤ 0.05 **; P-value ≤ 0.01; ***, P-value ≤ 0.001; ****, P-value ≤ 0.0001. **B.** Table demonstrating fold changes in biofilm production comparing the strains. (↓), decreased biofilm production; (↑) increased biofilm production. Results for pCMY-42 are in the same range as for pCMY-145.


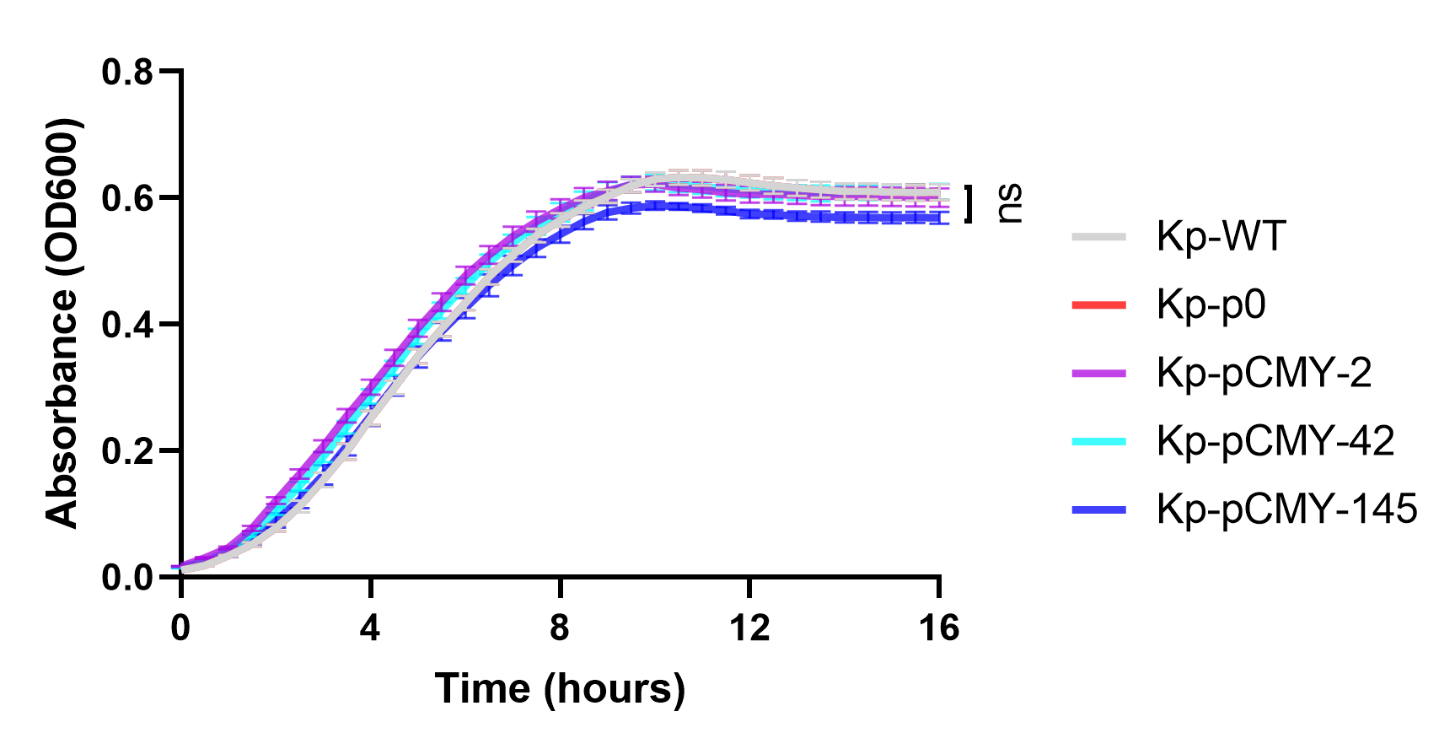


**Figure S7**. Measurement of growth capacities of the Kp-WT and recombinant isogenic *K. pneumoniae* isolates demonstrating no significant difference between the strains. Data is shown as mean ± SEM.


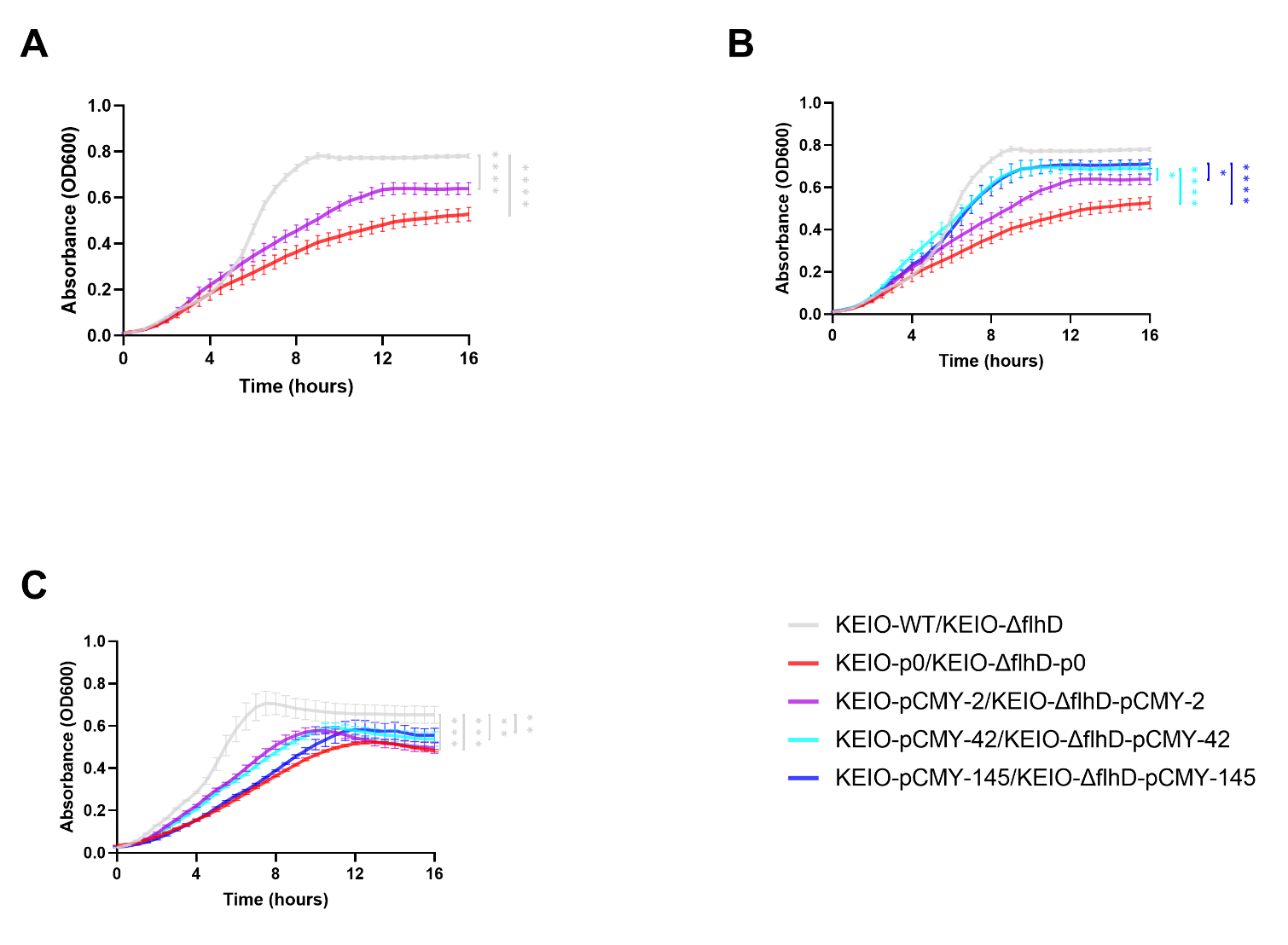


**Figure S8**. Measurement of growth capacities of the KEIO-WT and recombinant isogenic *E. coli* isolates. **A.** Growth curves evidencing the negative impact on the growth rate caused by the insertion of the plasmid. **B.** Demonstration of the effect of compensation observed when pCMY-42 and pCMY-145 are present. **C.** Demonstration of the increased growth rate observed for the KEIO-ΔflhD when compared with the recombinant strains and the lack of significant activity observed between KEIO-ΔflhD-pCMY-2 and KEIO-ΔflhD-pCMY-42 or KEIO-ΔflhD-pCMY-145. Data is shown as mean ± standard error of mean (SEM). *, P-value ≤ 0.05 **; P-value ≤ 0.01; ***, P-value ≤ 0.001; ****, P-value ≤ 0.0001.


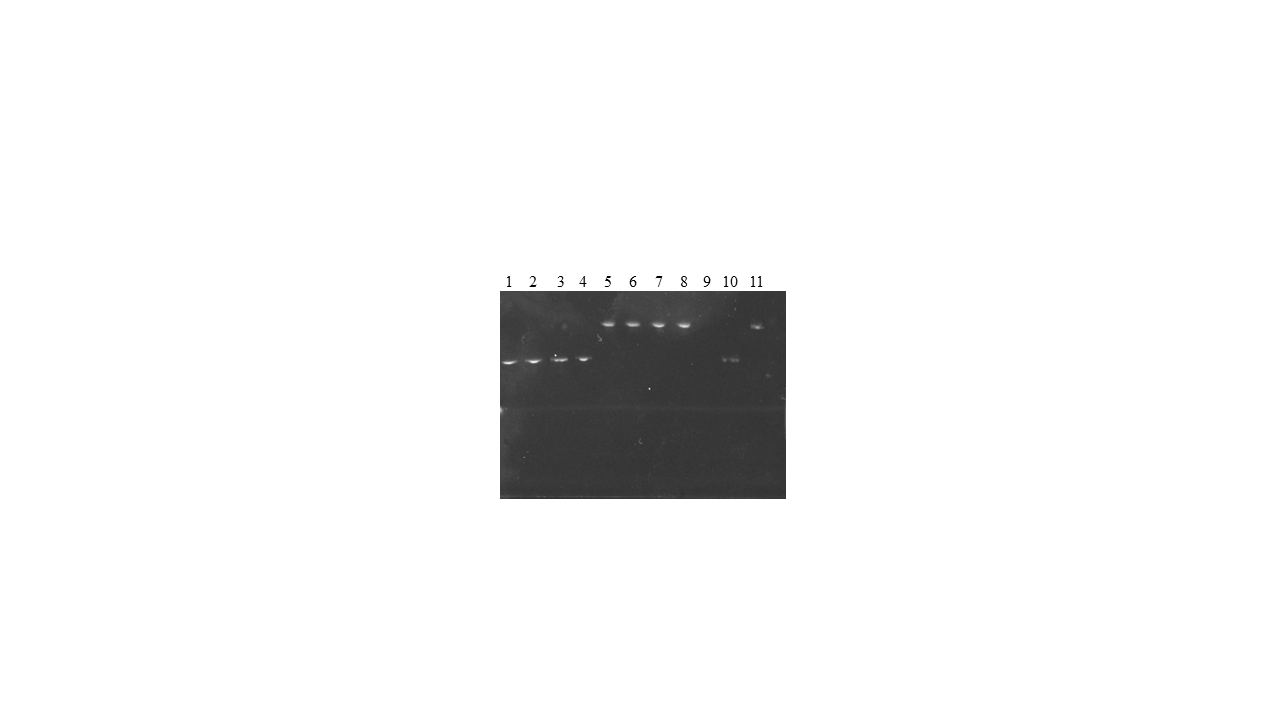


**Unpublished data 1**. Representative gel showing EMSA results stained with SYBR Green and visualized under UV radiation. Lane 1-4: *flhD* in 40 ng aliquots with increasing amounts of CMY-2 (65, 195, 390, and 780 ng). Lane 5-8: *flhD* plus its natural promoter in 40 ng aliquots with increasing amounts of CMY-2 (65, 195, 390, and 780 ng). Lane 9: negative control. Lane 10: *flhD* DNA only (40 ng). 11: *flhD* plus its natural promoter DNA only (40 ng). No changes in DNA size were observed in lanes 1-8.

**Table S1**. MICs of β-lactams demonstrating the loss of catalytic activity of CMY-145-Ser64Arg compared with pCMY-145.

| **Strain** | **Antibiotic** | | |
| --- | --- | --- | --- |
|  | **AMO** | **CTX** | **CAZ** |
| 25922 | 4 | 0,125 | 0,25 |
| MG1655 | 8 | 0,125 | 0,125 |
| MG1655+p0 | 8 | 0,125 | 0,125 |
| MG1655+pCMY-145 | 256 | 32 | 16 |
| MG1655+pCMY-145-Ser64Arg | 8 | 0,125 | 0,125 |

AMO, amoxicillin; CTX, cefotaxime; CAZ, ceftazidime

**Table S2**. Global correlations of transcriptomic analysis.

| **Correlations** | **Genes** | | |
| --- | --- | --- | --- |
|  | **Down-regulated** | **Up-regulated** | **Total** |
| pCMY-2 vs p0 | 327 | 473 | 800 |
| pCMY-42 vs p0 | 139 | 257 | 396 |
| pCMY-145 vs p0 | 144 | 320 | 464 |
| pCMY-42 vs pCMY-2 | 152 | 62 | 214 |
| pCMY-145 vs pCMY-2 | 88 | 23 | 111 |
| pCMY-145 vs pCMY-42 | 3 | 17 | 20 |

**Table S3**. Raw RNA sequence data showing the statistically significant gene expression differences when comparing p0, pCMY-2, pCMY-42, pCMY-145 (Excell table).

**Table S4**. *Zophobas morio* health index scoring system

| **Category** | **Description** | **Score** |
| --- | --- | --- |
| Activity | no movement, immobile | 0 |
|  | minimal movement on stimulation, reduced response | 1 |
|  | moderate movement, responds to stimuli | 2 |
|  | active movement, healthy behavior | 3 |
| Molting* | partial molting | 0 |
|  | no molting/full molting | 1 |
| Melanization | intense melanization, dark brown or black larvae | 0 |
|  | well-defined dark spots extending beyond the puncture site | 1 |
|  | slight melanization at the puncture site | 2 |
|  | puncture mark is visible with no melanization | 3 |
|  | no melanization | 4 |
| Survival | dead | 0 |
|  | alive | 2 |

*In Galleria mellonella, cocoon formation is one of the attributes but in ZmL does not occur. Molting is a suitable attribute that we have observed during infection experiments. Table adapted from Loh JM, Adenwalla N, Wiles S, Proft T. Galleria mellonella larvae as an infection model for group A streptococcus. Virulence. 2013 Jul 1;4(5):419-28. doi: 10.4161/viru.24930.

**Table S5.** Primers used for cloning into vector pTOPO, site directed mutagenesis experiments, RT-qPCR, cloning into vector pOPINF, and EMSA.

| **Primer** |  | **Sequence (5’-3’)** |
| --- | --- | --- |
| pTOPO-CMY-F |  | aacacactgattgcgtctgacg |
| pTOPO-CMY-R |  | ggcaaaatgcgcatgggatt |
| pTOPO-DHA-1-F |  | acacggaaggttaattctga |
| pTOPO-DHA-1-R |  | ttattccagtgcactcaaaatagcc |
| pTOPO-FOX-5-F |  | gacctcggaaatgtctcgga |
| pTOPO-FOX-5-R |  | cccggcgtaacagtcaaatt |
| pTOPO-ACC-1-F |  | tcttttgcatgcggattggc |
| pTOPO-ACC-1-R |  | agccaccgatctgttccttg |
| SDM_Ser64Arg-CMY-F |  | tgagctaggacgcgttagtaagacgtttaac |
| SDM_ Ser64Arg-CMY-R |  | aacagcgtttgctgc |
| SDM**_**Asn90Thr-CMY-F |  | taagacgtttaccggcgtgttgg |
| SDM_Asn90Thr-CMY-R |  | ctaaccgatcctagctcaaac |
| SDM_Val211Ser-DHA-F |  | accggtccgcagctcgccgggac |
| SDM_Val211Ser-DHA-R |  | tttttgtttttataaccgtacgc |
| SDM_Val211Ser-FOX-F |  | gcccgtccggagcactccgggcg |
| SDM_Val211Ser-FOX-R |  | ttatcttccttcgaatagccgtaggcatagttc |
| SDM_Val211Ser-ACC-F |  | gccagtgcacagcaatatggagattttg |
| SDM_Val211Ser-ACC-R |  | tcatctttcttgttgtagc |
| GFP_SmaI-F |  | gatgatcccggggattggatcgttggctcgtc |
| GFP-R_fseI-R |  | gatgatggccggccatgcctggaattaattccta |
| SDM_GFP-stop-F |  | taaaggagaataacttttcactgg |
| SDM-GFP-stop-R |  | ctcattttgtatagttcatcc |
| qPCR_*rpoD*-F |  | tgatgctggctgaaaacacc |
| qPCR_*rpoD*-R |  | agttcaacggtgcccatttc |
| qPCR_*flhC*-F |  | actttcaacaaaccgcacca |
| qPCR_*flhC*-R |  | ggtttgtgtaatggcgtcga |
| qPCR_*fliA*-F |  | taactgactgacccgcgatt |
| qPCR_*fliA*-R |  | tggaagccatcgaaacgttg |
| qPCR_*flgK*-F |  | accgacgaagcgaaaatagc |
| qPCR_*flgK*-R |  | tggcgctactggttttcaac |
| qPCR_*dsdA*-F |  | tcccgttttgcaccctatct |
| qPCR_*dsdA*-R |  | tttcagtaacagttgcccgc |
| qPCR_*gspC*-F |  | gcaaatcccgtggcagttaa |
| qPCR_*gspC*-R |  | gggcttcgttgagggagtat |
| fusion-CMY-F |  | ggtctggaaggtagcggtagcgccgcaaaaaca |
| fusion-CMY-R |  | ctggtctagaaagctttattgcagcttttcaagaatgcgc |
| EMSA-*flhD*-F-universal |  | agtgacaaaccagttgattg |
| EMSA-*flhD*-R |  | tgggaataatgcatacctcc |
| EMSA-*flhD*-R+promoter |  | gtcgatttaggaaaaatctt |

SDM, site directed mutagenesis
